# Silver Nanoparticles Anchored on Single-Walled Carbon Nanotubes via a Conjugated Polymer for Enhanced Sensing Applications

**DOI:** 10.1021/acsomega.3c01127

**Published:** 2023-04-05

**Authors:** Jianfu Ding, Zhao Li, Oltion Kodra, Martin Couillard, Jianying Ouyang, François Lapointe, Patrick R. L. Malenfant

**Affiliations:** † Security and Disruptive Technologies Research Centre, National Research Council of Canada, 1200 Montreal Road, M-12, Ottawa, Ontario K1A 0R6, Canada; ‡ Energy, Mining and Environment Research Centre, National Research Council of Canada, 1200 Montreal Road, M-12, Ottawa, Ontario K1A 0R6, Canada; § Security and Disruptive Technologies Research Centre, National Research Council of Canada, 1200 Montreal Road, M-50, Ottawa, Ontario K1A 0R6, Canada

## Abstract

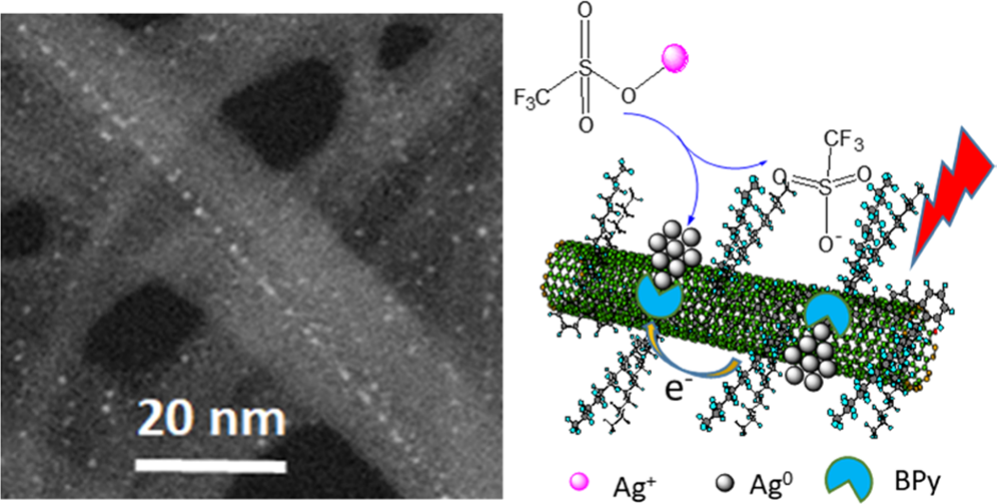

Single-walled carbon nanotubes (SWCNTs) are candidate matrices for loading metal nanoparticles (NPs) for sensor and catalytic applications owing to their high electron conductivity and mechanical strength, larger surface area, excellent chemical stability, and ease of surface modification. The performance of the formed NP/SWCNT composites is dependent on the NP size, the physical and chemical interactions between the components, and the charge transfer capabilities. Anchoring metal complexes onto the surface of SWCNTs through noncovalent interactions is a viable strategy for achieving high-level metal dispersion and high charge transfer capacities between metal NPs and SWCNTs. However, traditional metal complexes have small molecular sizes, and their noncovalent interactions with SWCNTs are limited to provide excellent sensing and catalytic capability with restricted efficiency and durability. Here, we selected poly(9,9-di-*n*-dodecylfluorenyl-2,7-diyl-*alt*-2,2′-bipyridine-5,5′) (PFBPy) to increase the noncovalent interactions between silver nanoparticles (AgNPs) and SWCNTs. A silver triflate (Ag–OTf) solution was added into a PFBPy-wrapped SWCNT solution to form Ag–PFBPy complexes on the nanotube surface, after which Ag^+^ was photoreduced to AgNPs to form a Ag–PFBPy/SWCNT composite in the solution. In various feeding molar ratios of Ag–OTf over the BPy unit (0.4–50), the size of the formed AgNPs may be well-controlled at sub-nm levels to provide them with an energy level comparable to that of the SWCNTs. Additionally, the 2,2′-bipyridine (BPy) unit of the polymer provided a coordinating interaction with Ag^+^ and the formed AgNPs as well. The 5,5′-linage of BPy with the fluorene unit in PFBPy ensured a straight main chain structure to retain strong π–π interactions with nanotubes before and after Ag^+^ chelation. All of these factors confirmed a tight contact between the formed AgNPs and SWCNTs, promoting the charge transfer between them and enhancing the sensing capabilities with a 5-fold increase in humidity sensing sensitivity.

## 1 Introduction

Owing to their excellent electron conductivity, mechanical strength, chemical stability, and large surface area,^1−3^ single-walled carbon nanotubes (SWCNTs) have attracted increasing attention as a matrix for loading metal nanoparticles (NPs) for catalysis^4−8^ and sensing applications.^9−13^ In these composite materials, the metal NPs provide specific active sites for the desired functionality, while the SWCNT networks provide robust mechanical support and efficient charge transport to enable the highly efficient transduction performance required for these applications.^4−13^ The NP/SWCNT nanocomposites can be prepared in two distinct ways. One method is depositing metal onto the SWCNTs using vacuum or solution deposition, where NPs adhere to the SWCNTs without a chemical bond.^14^ The other is to chemically connect them to the SWCNTs either covalently^15^ or noncovalently.^11−13^ This type of metal dispersion may be advantageous for sensing and catalytic applications with better performance and durability. A covalent linkage is typically formed by a chemical bond, such as -S- for Au,^3^ or by bonding a metal complex to the SWCNT surface.^16−18^ A noncovalent linkage can be produced by attaching a metal complex of a coplanar conjugated ligand, such as phthalocyanine, to the surface of SWCNTs.^11−13^ The π–π interactions between the ligand and SWCNTs provide an anchoring force for the metal on the SWCNT surface. In these systems, the sensing and catalytic capabilities rely on the charge transfer between the metal and the SWCNT network, which can be efficiently enhanced by a tight anchoring of the metal on SWCNTs.^19^ The anchoring capabilities based on the π–π interaction between the conjugated ligand and SWCNTs can be improved by employing a ligand with a large coplanar structure.^7−13^ It can generate stable metal/SWCNT composites with uniform metal coverage and high metal dispersion, even at an atomic level.^4,5^

However, due to the small size of this type of organic ligand, their π–π interaction strength with the nanotubes is restricted, and the formed metal complex/SWCNT composites for real-world applications are not highly robust and have a short lifetime.^5^ Incorporating functional groups, such as amines, into the organic ligand can strengthen the interactions between the ligand and SWCNTs and improve the stability of the composite.^4^ A chemically bonded complex/SWCNT system was also evaluated, in which the metal complex was linked to the SWCNT surface via a covalent bond on the ligand.^16^ However, this chemical bond typically degrades the close face-to-face packing of the complex on the SWCNT surface, resulting in decreased π–π interactions between the ligand and SWCNTs. Consequently, another strategy involving polymer wrapping was proposed,^20^ where a polymer with an organic ligand as the side chain was used to wrap the nanotubes. The formed Ag/pyridine complex in poly(4-vinylpyridine) was anchored to the nanotubes by polymer wrapping and exhibited excellent ammonia gas sensing capabilities.

**Scheme 1:**
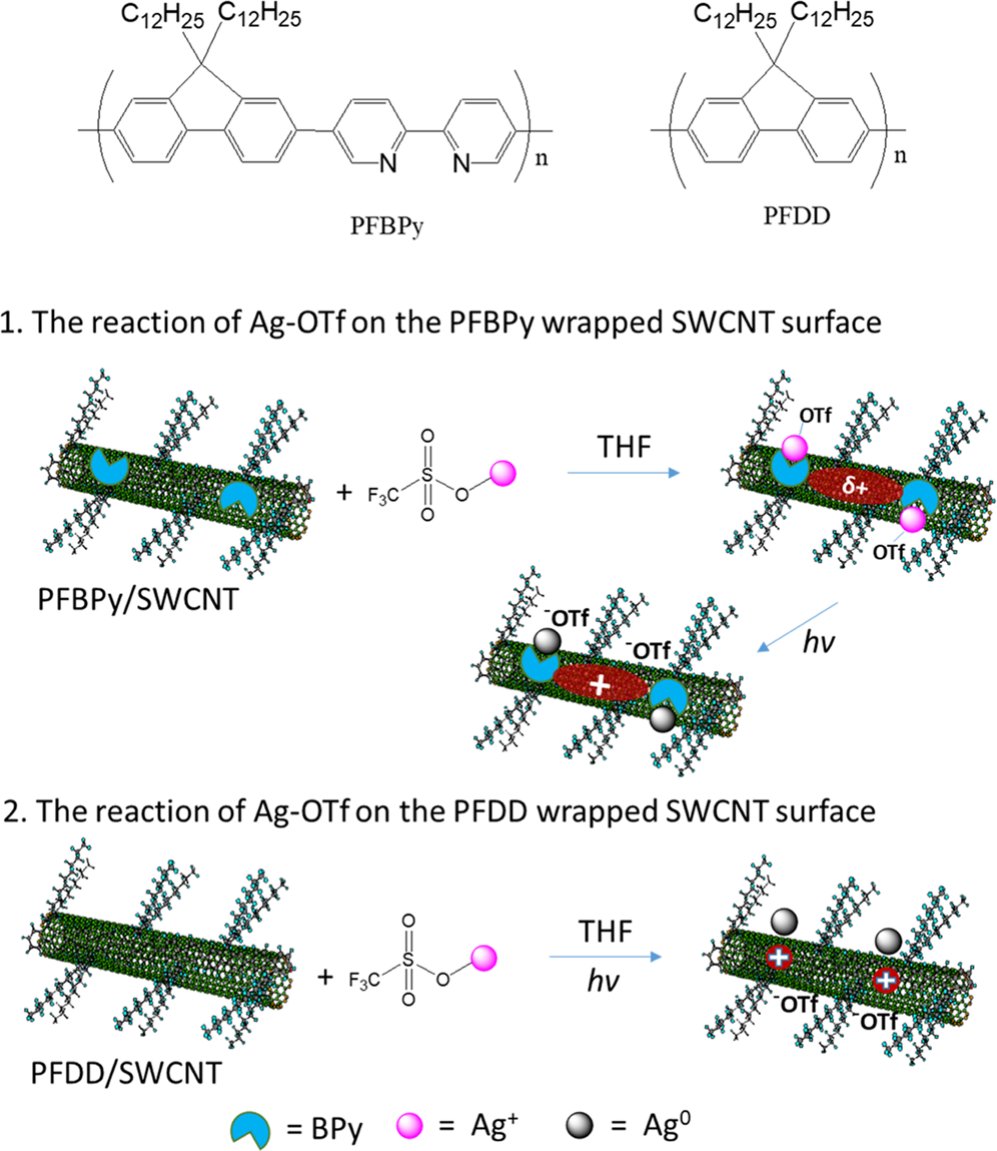
Chemical Structure of PFBPy and PFDD (top) and (1) the Coordinating Reaction of Ag–OTf with PFBPy on the SWCNT Surface, followed by the Reduction of the Chelated Ag^+^ to Ag^0^ by an Electron from the SWCNT upon Light Excitation. In this Material, PFBPy Provides a Strong Interaction with the Formed Ag^+^ to Anchor It on the SWCNT Surface through the Chelating Effect of the BPy Unit and the π–π Interactions of PFBPy with SWCNTs. (2) The Reaction of Ag–OTf on the PFDD Wrapped SWCNT Surface, Where Ag–OTf Undergoes a Simple Photocatalyzed Reduction without Any Anchoring Effect Due to the Missing Chelating Unit in the Wrapping Polymer

In this work, we introduced a new metal anchoring system using a conjugated polymer with a ligand in the conjugated main chain. In particular, we developed an alternating copolymer of fluorene with 2,2′-bipyridine (BPy), i.e., poly(9,9-di-*n*-dodecylfluorenyl-2,7-diyl-*alt*-2,2′-bipyridine-5,5′) (PFBPy); the structure is illustrated in Scheme 1. An analogous polymer with a short side chain has been reported and showed a specific ability to anchor metal onto SWCNTs.^21^ 2,2′-Bipyridine has been identified as a ligand with high electron transport and high redox stability.^22^ The 5,5′-linkage of the BPy unit with the fluorene comonomer ensures a fully conjugated structure of the polymer main chain. It will promote charge transfer between the anchored metal and SWCNTs in two ways. (1) The in-situ synthesis of AgNPs and the chelating interaction of Ag^+^ with BPy makes the formed NPs well-controlled at the sub-nm level so that they have the same energy level as the SWCNTs, generating an essential condition for efficient charge transfer. (2) The chelation of the metal with BPy in the polymer and the efficient π–π interaction between the large coplanar conjugated main chain of the polymer and the SWCNTs ensured a tight anchoring of the formed AgNPs on the SWCNT surface and a promoted charge transfer between them. The polymer conformational change during chelating with Ag^+^ is crucial in determining whether the strong polymer/SWCNT interactions are sustained following the reaction. For example, an analogous fluorene/BPy alternating copolymer with a 6,6′-bipyridine linkage exhibited a high capacity for sc-SWCNT enrichment, indicating strong π–π interactions with SWCNTs.^23,24^ However, a recent work showed that adding metal ions into this polymer-wrapped SWCNT solution stripped the polymer off the SWCNTs^25^ because of the metal coordinating forcing the BPy unit to adopt a cis-conformation, bending the polymer main chain to an angle of 60° at the BPy unit and damaging the compact packing of the polymer on the SWCNTs. PFBPy with a 5,5′-BPy linkage was used in our work to maintain the straight main chain structure upon complexation with the metal, ensuring a compact packing of the polymer and the strong π–π interactions with SWCNTs before and after the formation of the complex. Strong SWCNT interactions with a metal-complexed fluorene-phenanthroline supramolecular structure, successfully employed for sc-SWCNT enrichment, also demonstrated this effect.^26^

Ag–PFBPy/SWCNT composite solutions were prepared by dropping a Ag–OTf solution into a PFBPy/SWCNT solution. This reaction resulted in the formation of Ag–BPy complexes in the PFBPy polymer on the SWCNT surface. Additionally, room light has sufficient energy to excite electrons in SWCNTs and inject them into the Ag^+^ ions in the Ag–BPy complexes, and the formed Ag^0^ can serve as a seed for further Ag^+^ chelation and reduction to form AgNPs at the identical position. With BPy anchoring, Ag^+^ reduction is significantly faster than that without BPy units (PFDD/SWCNT), indicating a more efficient charge transfer between the metal and SWCNTs with the presence of a BPy conjugated polymer. Moreover, as demonstrated by high-resolution transmission electron microscopy (HRTEM), cyclic voltammetry (CV), and UV studies, this anchoring effect tightly fixed the formed AgNPs to the SWCNT surface, significantly improving the sensing properties of the produced composites with a 5-fold increase in detection sensitivity for humidity sensing in air.

## 2 Experimental Section

### 2.1 Samples

The PFDD/SWCNT composite was prepared from a laser SWCNT sample using a previously reported CPE method.^27,28^ The weight ratio of the polymer to SWCNTs in the CPE product was 1/1 and was adjusted to 2.5/1 in the final PFDD/SWCNT inks for this study. The PFBPy/SWCNT composite was prepared from the PFDD/SWCNT composite with a 1/1 weight ratio via a ligand exchange process; PFBPy with 10 times the SWCNT weight was mixed with the PFDD/SWCNT solution in toluene. The mixture was bath sonicated for 2 h,^29^ and was filtered to remove PFDD. By repeating this process one more time, the PFBPy/SWCNT composite with a weight ratio of 2.5/1 was obtained after the film from the filtration was thoroughly rinsed with the solvent (toluene) to remove the free polymer. The Ag–PFBPy/SWCNT composite was prepared by adding a Ag–OTf solution to this composite solution in THF to achieve a 0.4 [Ag]/[BPy] molar ratio or 0.0183 [Ag]/[CNT] ratio ([CNT], carbon molar concentration of SWCNT). Comparatively, a Ag–PFDD/SWCNT composite solution in THF was also prepared by adding the Ag–OTf solution to a 2.5/1 (weight ratio) PFDD/SWCNT solution at a [Ag]/[CNT] ratio of 0.0183. For the TEM investigation, a small drop of a diluted Ag–PFDD/SWCNT or Ag–PFBPy/SWCNT composite solution in THF was deposited on a 200-mesh TEM copper grid coated with a lacey carbon film, and the excess solution on the surface was immediately removed with a filter paper. For X-ray photoelectron spectroscopy (XPS) analysis, six film samples of PFDD, PFDD/SWCNT, Ag–PFDD/SWCNT (0.0183 of [Ag]/[CNT]) and PFBPy, PFBPy/SWCNT, Ag–PFBPy/SWCNT (0.0183 of [Ag]/[CNT]) were coated onto an aluminum strip by drop casting the corresponding solutions in THF. After coating, the samples were heated at 130 °C for 30 min to promote solvent evaporation.

### 2.2 Absorption Spectrum

The absorption spectra of the polymers, polymer/SWCNT composites, and their Ag–polymer/SWCNT composite solutions in THF were collected on a UV–Vis–NIR spectrometer (Cary-5000) in a range from 200 to 3200 nm.

### 2.3 TEM

An FEI Titan 80–300 TEM operated at 300 keV and equipped with a CEOS aberration corrector for the probe-forming lens and a monochromatic field-emission gun were used to obtain HRTEM and annular dark-field (ADF) images.^30^ HRTEM provided higher contrast for imaging carbon nanotubes and polymers and was used to examine the polymer/SWCNT composite samples. In scanning transmission electron microscopy (STEM) mode, ADF images were collected using a high-angle annular dark-field Fischione detector. This technique provides signal intensities mostly related to the atomic number (*Z*) and thickness of the investigated region. When combined with an aberration corrector, ADF-STEM can attain sub-Angstrom resolutions and single-atom sensitivity, and it was used to scan Ag atoms and NPs.

### 2.4 XPS

X-ray photoelectron spectroscopy (XPS) analyses were conducted using a Kratos Axis Ultra DLD XPS with a monochromatic Al Kα X-ray source (12 mA, 15 kV) and an analysis area of 300 μm × 700 μm. XPS can detect all elements except hydrogen and helium to a depth of 5–7 nm and has detection limits ranging from 0.1 to 0.5 atom % depending on the element. A Kratos charge neutralizer system was used on all specimens. Survey scan analyses (pass energy of 160 eV) were conducted at three different spots on each sample to check for uniformity, and the spectra were averaged to improve the S/N ratio. High-resolution analyses were performed on a single spot in each sample (energy of 160 eV). The spectra were corrected to the main line of the C 1s spectrum (polymeric carbon) set at 285.0 eV and were analyzed using Casa XPS software. The instrument resolution is 0.4 eV. Under this resolution, the instrument uncertainty of the peak energy for Ag 3d_5/2_ is lower than 0.02 eV.^31^ Therefore, the uncertainty of the peak position is largely dependent on the C 1s calibration.

### 2.5 CV

The CV measurement was performed in acetonitrile using a Solartron SI 1287 potentiostat with a gastight cell, at a scan rate of 50 mV/s and at 20 °C.^32^ A three-electrode configuration was used with a Ag wire serving as the quasi-reference electrode, a platinum (Pt) wire as the counter electrode, and a platinum disk of 1 mm diameter encased in a soft glass rod as the working electrode. The sample was coated on the working electrode by applying a small drop of solution (∼0.5 μL). After drying, the coated electrode was heated at 80 °C for 1 min and placed in the cell alongside the counter and quasi-reference electrode. The cell was also loaded with tetrabutylammonium hexafluorophosphate (Bu_4_NPF_6_, Fluka, electrochemical grade) and vacuum-dried at 80 °C for 20 min. Then, ∼4 mL of acetonitrile (HPLC grade) was distilled (over CaH_2_) into the cell under vacuum to produce a 0.1 M Bu_4_NPF_6_ solution. The CV curves were recorded by scanning potentials against the Ag quasi-reference electrode.

### 2.6 Thin-Film Transistor (TFT) and Sensor Test

The TFT test was conducted using Fraunhofer chips of 4 × 4 devices with four different channel lengths (2.5, 5.0, 10, and 20 μm) and a channel width of 1 mm. The active layer was coated by applying a drop of a THF solution in the solvent atmosphere. The excess solution was drained after 10 min, and the film was annealed overnight at 200 °C in a N_2_ glovebox to remove any residual moisture and oxygen. The test was conducted by scanning the gate voltage from −10 to 10 V, and the *I*–*V* curve was recorded in the glovebox. The sensing device was prepared by coating the sample solution by following the same procedure as for the TFT test but on an Ossila chip containing five identical TFT devices with a 30-μm channel length and a 1-mm channel width in an interdigitated configuration. The humidity sensor test was conducted in the sensor testing chamber under a chemiresistor mode with the setup illustrated in Figure S1. During the test, dry air from a cylinder was connected to an RH-200 Relative Humidity Generator (L&C Science and Technology). A constant flow of 250 sccm air at 50% RH was introduced into the chamber controlled by a mass flow controller as the carrier gas. A dry air pulse of 10 s followed by a 60 s pause was introduced into the sensing chamber by another mass flow controller at flow rates of 10, 20, 30, 40, and 50 sccm, corresponding to corrected RH levels of 48.1, 46.3, 44.6, 43.1, and 41.7%, respectively.

## 3 Results and Discussion

### 3.1 Humidity Sensing Capability of Ag–PFBPy/SWCNT Composites

**Figure 1:**
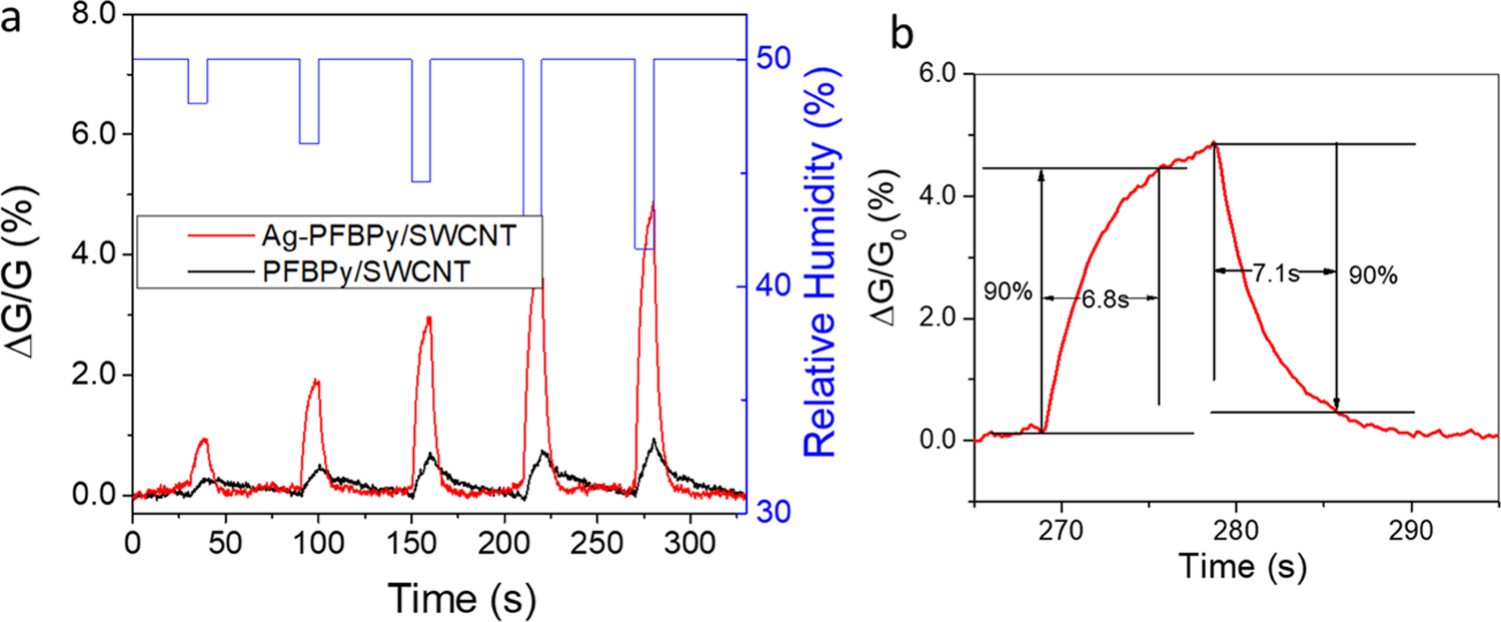
Sensing profile of chemiresistors for moisture sensing in air at 50% RH. (a) Comparison of the response (Δ*G*/*G*_0_) curve of the Ag/PFBPy/SWCNT and PFBPy/SWCNT devices to an air sequence with a lower RH (48.1, 46.3, 44.6, 43.1, and 41.7%). (b) The sensing profile of the Ag/PFBPy/SWCNT device with the RH changed from 50 to 41.7%, which gives the response and recovery times of 6.8 and 7.1 s, respectively, and the control device showed response and recovery times of 9 and 37 s, respectively.

To check the advantage of this type of silver-doped SWCNT composite for sensing applications, the humidity sensing behavior of a Ag–PFBPy/SWCNT chemiresistor at ∼50% RH was compared with that of a control device^33,34^ comprised of the same composite material but without silver. Figure 1 compares their response curves to an air pulse sequence with lower RHs (48.1, 46.3, 44.6, 43.1, and 41.7%). The device response [Δ*G*/*G*_0_ (%)] was recorded as an increase in conductance according to eq 1.
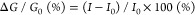
1where *I*_0_ and *I* denote the current of the sensor before and after exposure to the dryer air pulse. Figure 1 shows that the Ag–PFBPy/SWCNT device had an approximately 5-time increase in response compared to the device without silver, where only 1% response was observed when the RH decreased from 50 to 41.7%. This value is consistent with the results reported for semiconducting carbon nanotube devices^33^ in which the difference in the sensing response between 40 and 50% RH air was ∼2%. Our results indicated an apparent enhancement of the sensing response by introducing silver into this composite. Similar work has recently reported enhanced response and recovery times (15 s) by introducing AgNPs onto another carbon semiconducting material (graphene quantum dots).^34^ In our work, Figure 1b shows better response and recovery times of 6.8 and 7.1 s, respectively, which are likewise significantly lower than those of the control device (9 and 37 s), and those of many other nanomaterial-based humidity sensors.^34^ To better understand this improvement in humidity sensing by incorporating silver into the PFBPy/SWCNT composite material, we must investigate the reaction when Ag–OTf was introduced into this composite solution, the chemical and morphological structure, and the interactions between each component of the formed materials.

### 3.2 Absorption Spectroscopic Study

**Figure 2:**
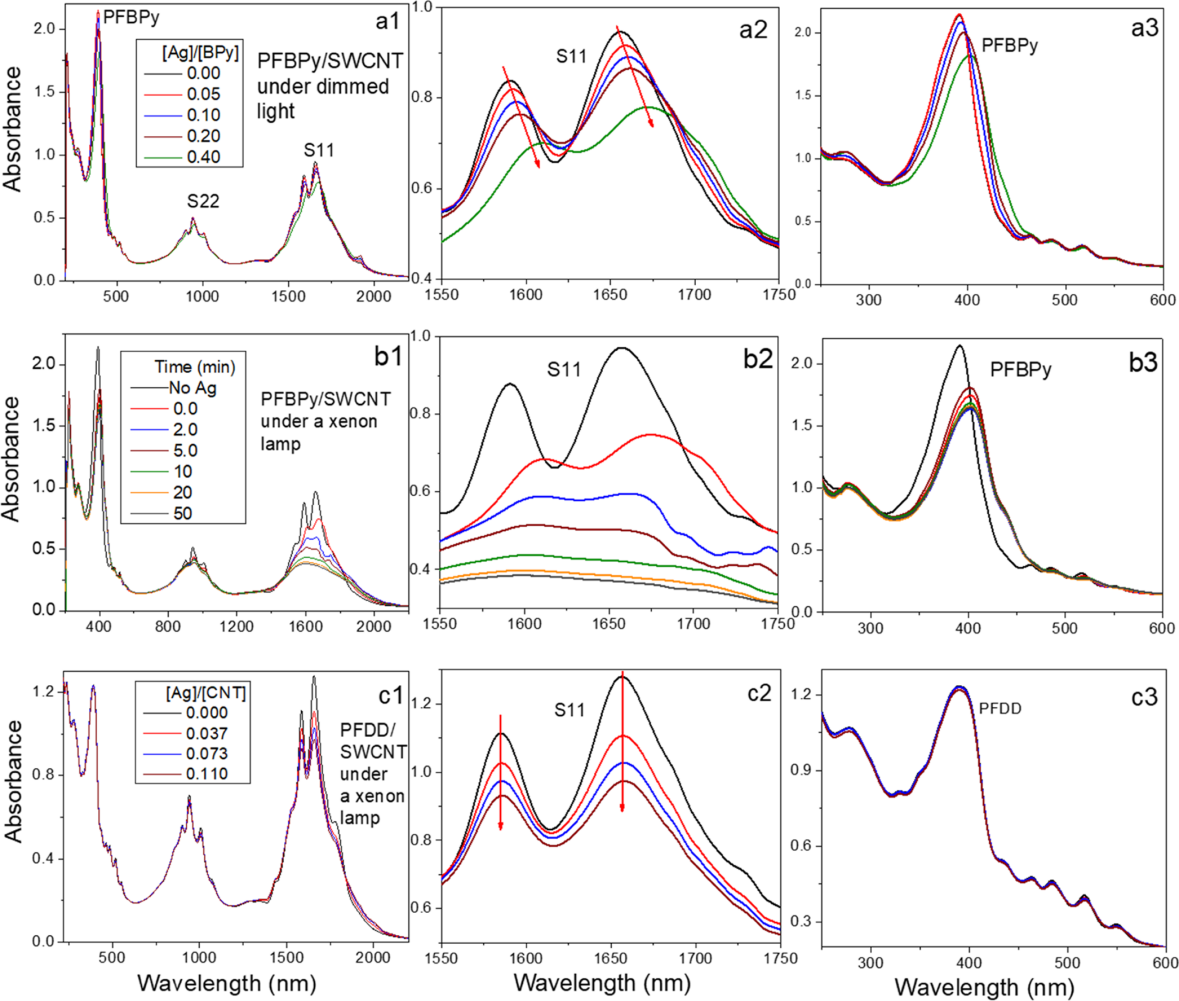
(a) Variation of the absorption spectrum of PFBPy/SWCNT in THF solution during the titration of Ag–OTf under dim lighting. The absorption spectrum of the titrated solution was collected at the [Ag]/[BPy] ratio of 0.00, 0.05, 0.10, 0.20, and 0.40 (or 0.000, 0.0023, 0.0046, 0.0, 0.0092, and 0.0183 of [Ag]/[CNT]). (b) The last solution of (a) at a [Ag]/[BPy] ratio of 0.40 was irradiated under a Xenon lamp at 110 W/m^2^ for 2, 5, 10, 20, and 50 min. (c) Ag–OTf titration to the PFDD/SWCNT solution (at 1/1 PFDD/SWCNT weight ratio) at [Ag]/[CNT] of 0.036, 0.073, and 0.11 under a Xenon lamp at an intensity of 110 W/m^2^ for 10 min.

**Figure 3:**
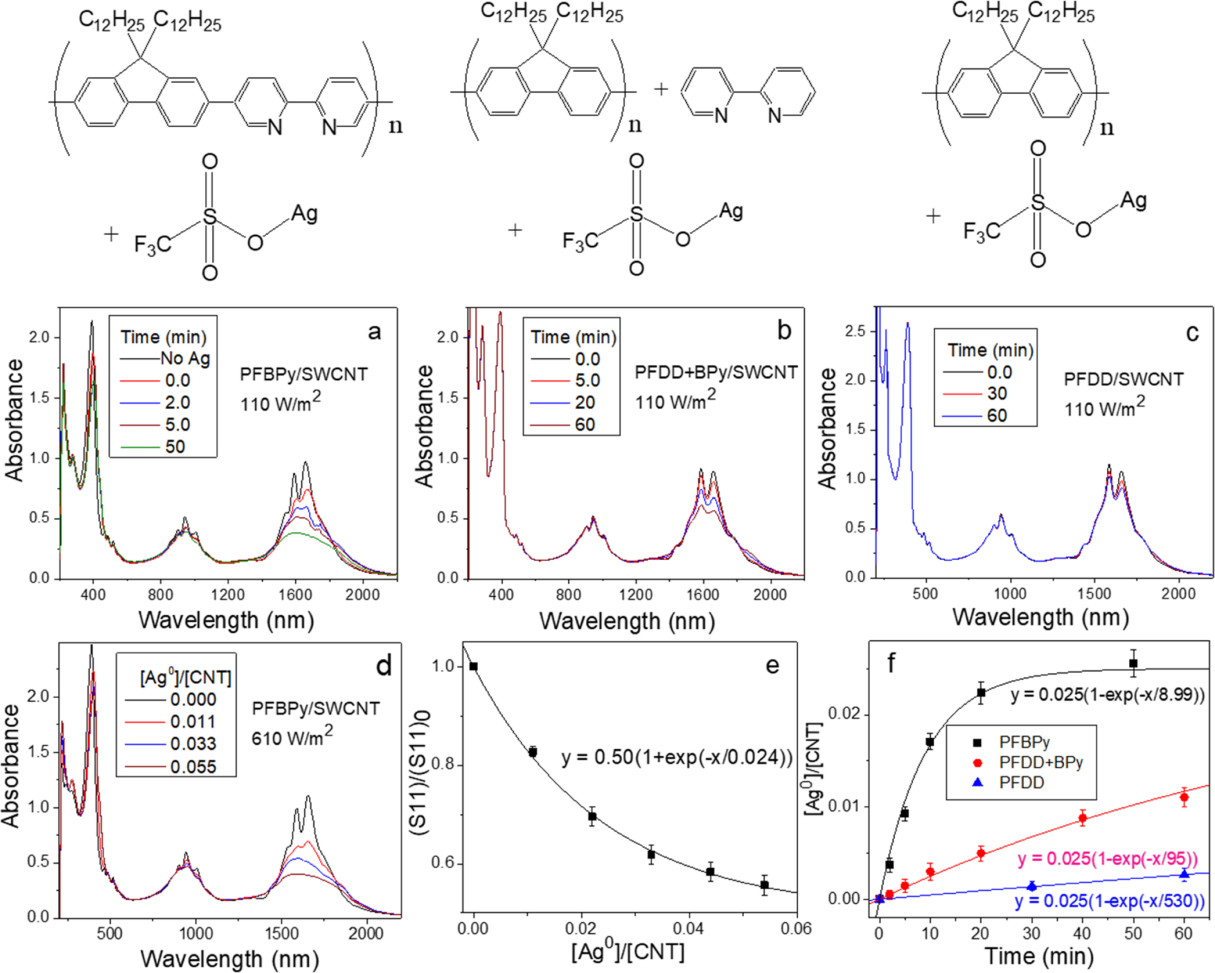
Chemical structure of the polymer and other reactants in the SWCNTs composite solution (top) and the variation of the absorption spectrum with the light irradiation (110 W/m^2^, corresponding to room sunlight) time of the polymer/SWCNT composite solution in THF with the P/CNT weight ratio of 2.5/1 and the addition of Ag–OTf at 0.0183 of [Ag]/[CNT] (0.4 of [Ag]/[BPy]) for (a) PFBPy/SWCNT, (b) PFDD + BPy/SWCNT, and (c) PFDD/SWCNT. (d) Variation in the absorption spectrum of the PFBPy/SWCNT solution with the addition of different amounts of Ag–OTf under a strong light (610 W/m^2^) for 8 min, (e) the extracted [Ag^0^]/[CNT] value plotted against (S11)/(S11)_0_ with the best fitting curve, which was used as a calibration for the Ag conversion analysis. (f) The calculated [Ag^0^]/[CNT] using this calibration at different irradiation times of the light (110 W/m^2^) was plotted for the three Ag–OTf doping solutions.

The interaction of Ag–OTf with the polymer and SWCNTs in the PFBPy/SWCNT and PFDD/SWCNT solutions was monitored by studying the variation in their absorption spectra during titration with the Ag–OTf solution. Figure 2a depicts the PFBPy/SWCNT absorption spectra after the Ag–OTf solution was added to the PFBPy/SWCNT solution under dim lighting. Three major absorption bands were observed in the entire range from 200 to 2200 nm, i.e., PFBPy peak at ∼390 nm, S22 band, and S11 band of SWCNTs at ∼938 and ∼1650 nm, respectively. Due to the complex chirality of the nanotubes, the S11 and S22 bands are composed of several peaks. During Ag–OTf titration, the polymer absorption peak and S11 and S22 bands of the SWCNTs displayed redshifts with the increase of Ag usage (Figure 2a2,a3). The redshift of the polymer peak indicates an increase in the effective conjugation length of the polymer main chain. This is associated with a higher coplanar conformation of the BPy unit in the polymer upon coordinating with Ag^+^.^35,36^ In conjugation with the peak redshift, the S11 and S22 bands widened slightly as the peak maximum decreased, but the overall peak intensity remained the same as the Ag–OTf content increased. This redshift of the PFBPy/SWCNT solution is attributed to the change in the local dielectric screening effect caused by the interaction of Ag^+^ with PFBPy on the SWCNT surface.^37^ As indicated by the redshift of the PFBPy absorption peak, the Ag–BPy complex was formed immediately upon the addition of Ag–OTf. It will increase the dielectric constant of the polymer layer on the nanotubes, resulting in a redshift of the S11 and S22 peaks. With the addition of 0.4 equiv of Ag^+^, the S11 peak shifts significantly (16 nm), indicating that most of the formed Ag–PFBPy complex is on the nanotube surface. Without the BPy unit in the polymer, adding the same amount of Ag–OTf to the PFDD/SWCNT solution under dim lighting did not alter the S11 and S22 absorption (not shown here). Only when the PFDD/SWCNT solution was irradiated with 110 W/m^2^ light for 10 min, a large peak intensity decrease occurred without any peak shift (Figure 2c). This change is attributed to SWCNT doping by the photocatalyzed Ag^+^ reduction,^6^ as will be discussed later. This reaction converted Ag^+^ to Ag^0^ in the solution, leaving a hole in the nanotube. This process will heavily p-dope the nanotubes, resulting in a decrease of the S11 and S22 peaks. This process was also observed in the Ag^+^-doped PFBPy/SCWNT solution, as depicted in Figure 2b, where the change in the absorption spectrum was recorded when the final Ag^+^-doped PFBPy/SCWNT solution from Figure 2a was exposed to 110 W/m^2^ light for varying amounts of time. As evidenced by the drastic fall in peak intensity and restoration of the peak position to its original position, a considerably efficient p-doping occurred during light irradiation. This process simultaneously released BPy from the complex and reduced the dielectric screening effect of the encapsulating polymer to reduce the peak redshift gradually. To better understand this difference, the reaction kinetics of these two reactions with 0.4 equiv Ag–OTf under the same irradiation (110 W/m^2^), as well as with the PFDD/SWCNT solution including an equivalent (vs fluorene unit) of 2,2-bipyridine (BPy) to mimic the PFBPy composition (the detailed reaction speed is discussed in Section 2 of the SI), was compared (Figure 3). It shows that the Ag^+^ reduction in the PFBPy/SWCNT composite solution is 59 times faster than that in the PFDD/SWCNT solution. However, when the BPy unit was isolated from the polymer, i.e., an equivalent BPy small molecule was mixed with the PFDD/SWCNT solution to mimic the PFBPy/SWCNT composition, the reaction speed only increased ∼5.6 times or ∼10% of that in the PFBPy/SWCNT solution, indicating a signification effect of PFBPy in promoting this Ag^+^ reduction.

**Scheme 2:**
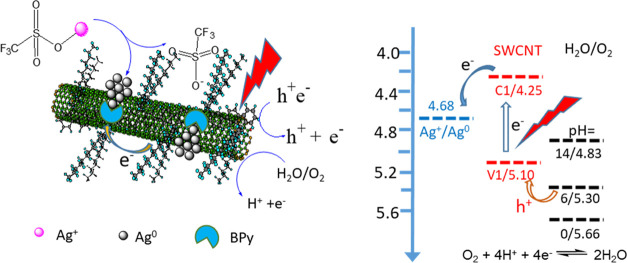
Reaction Mechanism for Ag Reduction on SWCNTs in Air under Light Irradiation: When Ag–OTf was Added to the PFBPy/SWCNT Composite Solution, the Ag^+^ Ion Chelated with the BPy Unit of the Polymer to Form a Complex. Then, an Excited Electron Generated by the Irradiation of the SWCNT was Injected into this Anchored Ag^+^. The Reduced Ag^0^ Domains Served as Seeds for Additional Ag^+^ Deposition and Reduction to Form AgNPs. This Process Generated Excess Holes (h^+^) in the Nanotube, Which Might Be Neutralized by ^–^OTf Anion, and the Hole Concentration Will Be Regulated Further by the H_2_O/O_2_ Redox Pair^39^

As illustrated in Scheme 2, this phenomenon depicted in Figure 2 is associated with the formation of AgNPs under light irradiation, where the light excited the highest occupied molecular orbital electron of SWCNTs to the lowest unoccupied molecular orbital, with sufficient energy to reduce Ag^+^ adsorbed on the nanotube surface, and the Ag^+^ reduction may start as
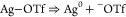
2In this reaction, the electron from SWCNTs reduced Ag^+^ to Ag^0^. This process will release the BPy ligand in the PFBPy, permitting a second Ag^+^ to bind and be reduced at the exact location. This process will be repeated to increase Ag^0^ deposition and produce AgNPs. According to the subsequent description of the TEM investigation, AgNPs dominate the sample, indicating that the reduced Ag^0^ site is favorable for further Ag^+^ binding and reduction. It may benefit from the low work function of the extremely small AgNP,^38^ which provides a large overpotential for extracting electrons from SWCNT. In addition, the tight anchoring of AgNPs on the SWCNTs ensured by the strong π–π interaction generated by the PFBPy polymer is essential for efficient Ag^+^ photoreduction. Without incorporating the BPy into the conjugated polymer, the Ag^+^ reduction in the PFDD + BPy/SWCNT solution is ∼10 times slower (Figure 3f).

### 3.3 CV Measurements

**Figure 4:**
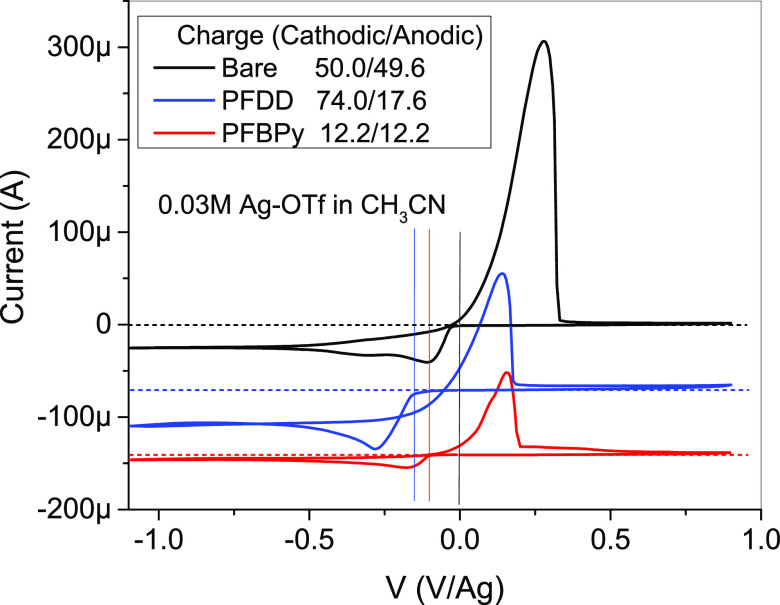
First scan of CV curves of 0.03 M Ag–OTf solution in CH_3_CN containing 0.1 M Bu_4_NPF_6_ on an uncoated, PFDD/SWCNT- and PFBPy/SWCNT-coated Pt electrode with a scan sequence from 0 to −1.1 to 0.9 and to 0 V vs Ag reference electrode. The curves for PFDD and PFBPy are downshifted for 70 μA, and a 0 A current line (dot line) was associated with each curve for a clear indication.

The interactions between Ag, the polymer, and SWCNTs in Ag–PFDD/SWCNT and Ag–PFBPy/SWCNT composite samples were studied using CV measurements under the following two experimental designs. (1) The influence of the coating layer was investigated by comparing the Ag^+^ redox behavior of the Ag–OTf solution on an uncoated, PFDD/SWCNT-coated, and PFBPy/SWCNT-coated Pt working electrode. (2) The redox behavior of the series of PFDD and PFBPY samples (polymer, polymer/SWCNT, and Ag–polymer/SWCNT) was compared by coating the corresponding materials on the Pt working electrode. Figure 4 displays the first CV scan of the Ag–OTf solution (0.03 M) with a scan sequence from 0 to −1.1 to 0.9 and back to 0 V relative to a Ag reference electrode. On all three electrodes, the forward scan reveals a Ag^+^ reduction wave followed by an oxidation wave in the reverse scan. This process was reversible and composed of a weak but broad reduction wave and a large and sharp oxidation wave at all three electrodes, showing that Ag^+^ reduction is a diffusion-controlled process and that the formed Ag^0^ deposit can be rapidly oxidized to Ag^+^ during the oxidation scan. Figure 4 also shows that the turn-on voltages of the reduction process on the PFBPy/SWCNT- and PFDD/SWCNT-coated electrodes were delayed by 0.1 V and 0.15 V, respectively, showing that the coating layers had a screening effect. Additionally, the reduction current on the PFDD/SWCNT electrode was ∼50% stronger than that on the uncoated electrode, attributed to the larger surface area of the SWCNT layer for Ag^+^ reduction. But the reduction current on the PFBPy/SWCNT electrode was ∼24% of the uncoated electrode, indicating that the amount of Ag^+^ on the electrode was regulated by the total amount of BPy units. The charge for reduction and oxidation can be determined by integrating the CV curves against time rather than voltage. The results indicated a balanced reduction/oxidation process on the uncoated and PFBPy/SWCNT-coated electrodes. However, the oxidation charge on the PFDD/SWCNT-coated electrode was only 24% of the reduction charge, indicating that the majority of the formed Ag^0^ deposit was inaccessible for the subsequent oxidation, i.e., a considerable proportion of the formed Ag particles only have loose contact with SWCNTs. This difference between the PFDD and PFBPy electrode highlights the crucial role of PFBPy in establishing a tight contact between the formed AgNPs and SWCNTs to ensure an efficient charge transfer, whereas the PFDD/SWCNT composite lacks this characteristic.

**Figure 5:**
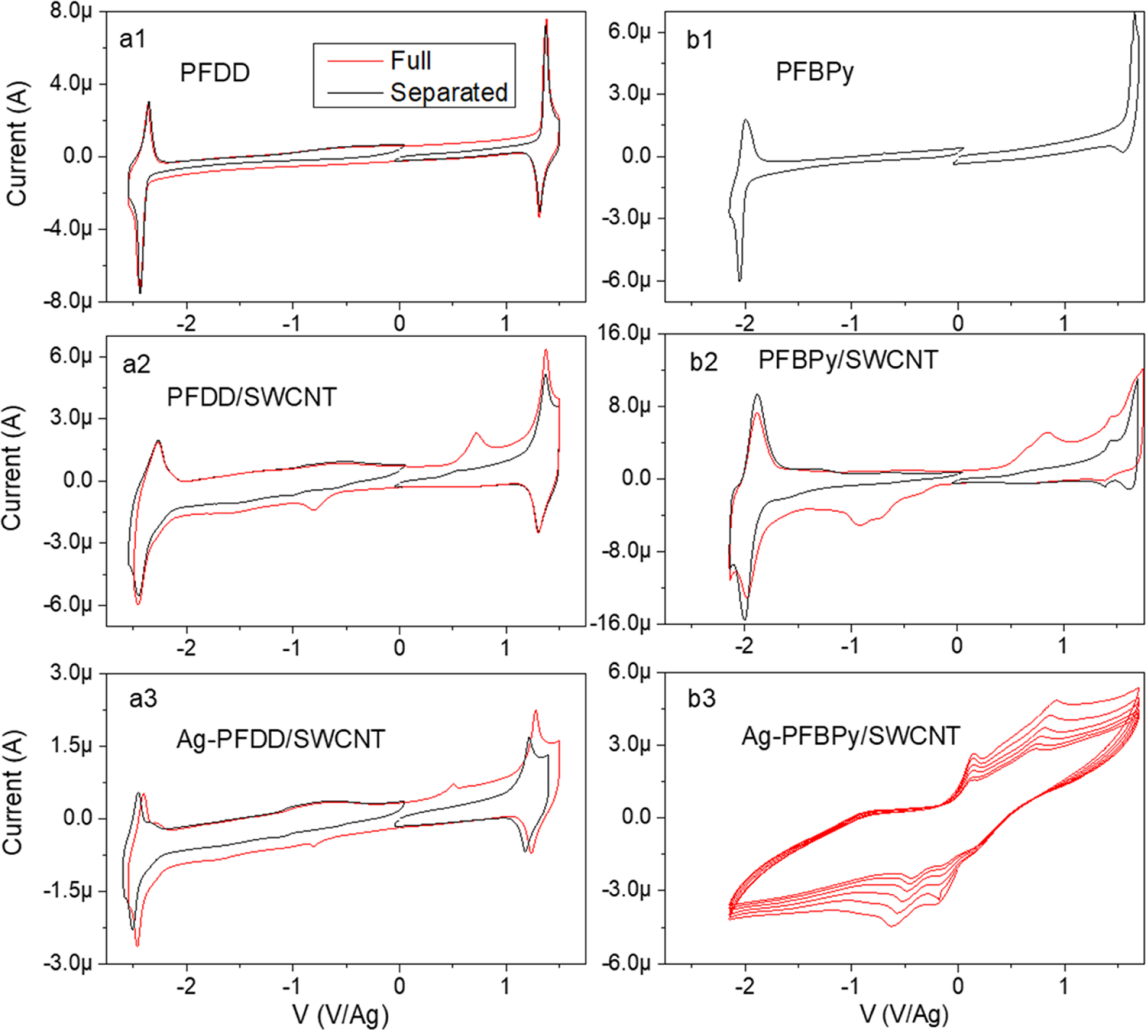
Cyclic voltammograms of the series of PFDD (a1–a3) and PFBPy (b1–b3) samples on a Pt electrode. Each series includes the pure polymer, polymer/SWCNT composite, and Ag–polymer/SWCNT composite. The black curves are from the second scan, where the reduction and oxidation processes were scanned separately, and the red curves are from the second scan over the entire reduction and oxidation region. The [Ag]/[CNT] molar ratio in the Ag–PFBPy/SWCNT composite was 0.0183, corresponding to [Ag]/[BPy] = 0.4/1, and the same [Ag]/[CNT] ratio was used for the Ag–PFDD/SWCNT composite.

Then, the CV behavior of the materials at different compositing phases, i.e., polymer, polymer/SWCNT, and Ag–polymer/SWCNT was investigated by coating the related materials on the Pt working electrode. We examined the CV curves from the 2nd and subsequent scans because the initial scan curve is affected by the doping history of the material. Figure 5 compares the CV curves under two scan modes, i.e., scan in the overall range for reduction and oxidation (red curve) and in the separated reduction or oxidation range (black curves), and Figure 5a1 shows that the pure PFDD film has a highly reversible reduction and oxidation process for both the overall range scan and the separated reduction or oxidation scans, indicating high stability of this polymer for both negative and positive charging. Figure 5a2 (red curve) displays that as SWCNTs were incorporated into the polymer, a small additional peak appeared at −0.79 V in the reduction scan with an onset at −0.50 V, a counter peak appeared in the reverse scan at +0.7 V with an onset at 0.35 V for the whole range scan, and this feature was retained in the subsequent scans (not shown here). However, these two small peaks disappeared in the second and subsequent scans when the sample was scanned separately in the oxidation or reduction range (black curve). For the separated scans, a comprehensive study revealed that these two peaks only appeared in the first scan after the sample underwent the opposite charging process, i.e., they appeared in the first scan of the reduction CV scan immediately after the sample underwent an oxidation scan or vice versa. These two new peaks are assumed to be associated with the reduction and oxidation of the SWCNTs in the composite.^40^ The SWCNTs used are laser tubes with diameters between 1.2 and 1.4 nm.^28,41^ The band gap of 0.85 eV calculated from the onset data is consistent with the predicted band gap value.^39^ However, Figure 5a2 shows only charge injection peaks for both reduction and oxidation but no charge release peaks. This can be explained by charge trapping.^42^ During the CV scan, SWCNTs can hold the injected charges in the SWCNT/polymer composite, and they can only be neutralized during the reverse redox process when the opposite charges are injected. This also explains why the charge injection peak only appeared in the initial scan when the sample was scanned independently for oxidation and reduction. In this case, the SWCNTs were saturated with charge after the first charge scan because the scan is limited in oxidation or reduction range, and no additional charge could be injected during subsequent scans.

After Ag–OTf was added to this composite, the CV curve in Figure 5a3 resembles that of the sample without Ag–OTf, displaying a definite charge injection peak for SWCNT reduction and oxidation at the same position as in Figure 5a2. However, no Ag redox peaks are observed for the Ag–PFDD/SWCNT composite. Although most of Ag^+^ was photoreduced to AgNPs on SWCNTs in this sample, the AgNPs have loose contact with SWCNT, as evidenced by the CV result in Figure 4, which is based on weak van der Waals interactions, resulting in low synergistic effects.^43^ Furthermore, for the portion of AgNPs that have good contact with SWCNTs, as soon as they were oxidized to Ag^+^ during the initial oxidation scan, they can migrate into the electrolyte solution. Due to the relatively large volume of the electrolyte solution (∼4 mL) and the small size of the coated film (∼1 × 10^–7^ mL), the Ag^+^ concentration could have been diluted by a factor of ∼10^8^, which explains why no Ag^+^/Ag^0^ redox pair was detected in the second and subsequent CV scans. In contrast, a distinct Ag redox pair with nearly symmetric reduction and oxidation peaks was observed for the Ag–PFBPy/SWCNT composite and this feature was highly reproduced in the successive scans, as shown in Figure 5b3, indicating that the AgNPs in this composite could easily be oxidized and then reduced during the CV scans. This finding demonstrated that the BPy units in PFBPy may effectively hold the formed Ag^+^ for subsequent reduction on-site. Furthermore, we also sonicated this Ag–PFBPy/SWCNT solution for 10 min and re-evaluated this CV behavior. Sonication does not impair the adhesion of AgNPs to SWCNTs, as evidenced by the fact that a fully reproducible result was obtained in the CV study. This result demonstrates a significant improvement in the robustness of the Ag/SWCNT composite by the use of PFBPy to anchor silver on the nanotube surface upon redox reaction, thus enhancing the charge transfer process between them. This feature is advantageous for using this material for catalysis and sensing applications. Figure 5b1,b2 depicts the CV behaviors of pure PFBPy and its SWCNT composite, which are remarkably comparable to those of the PFDD counterpart except for two observable differences: (1) Both the reduction wave and oxidation wave of the polymer have an approximately 0.45 V positive shift, indicating that they accept electrons more readily than PFDD due to the presence of BPy unit in the polymer. (2) The partially reversible oxidation waves of the polymer indicate that the positively charged state of this polymer is less stable. Figure 5b2 reveals a double mode of the SWCNT cathodic peak for both the reduction and oxidation processes, with the major peaks shifted outward by ∼ 0.1 V relative to Figure 5a2 for PFDD/SWCNT. It is possible that some of the injected charges in this material were stabilized by the dielectric effect of the polar BPy units in the wrapped PFBPy.

### 3.4 XPS Study

**Figure 6:**
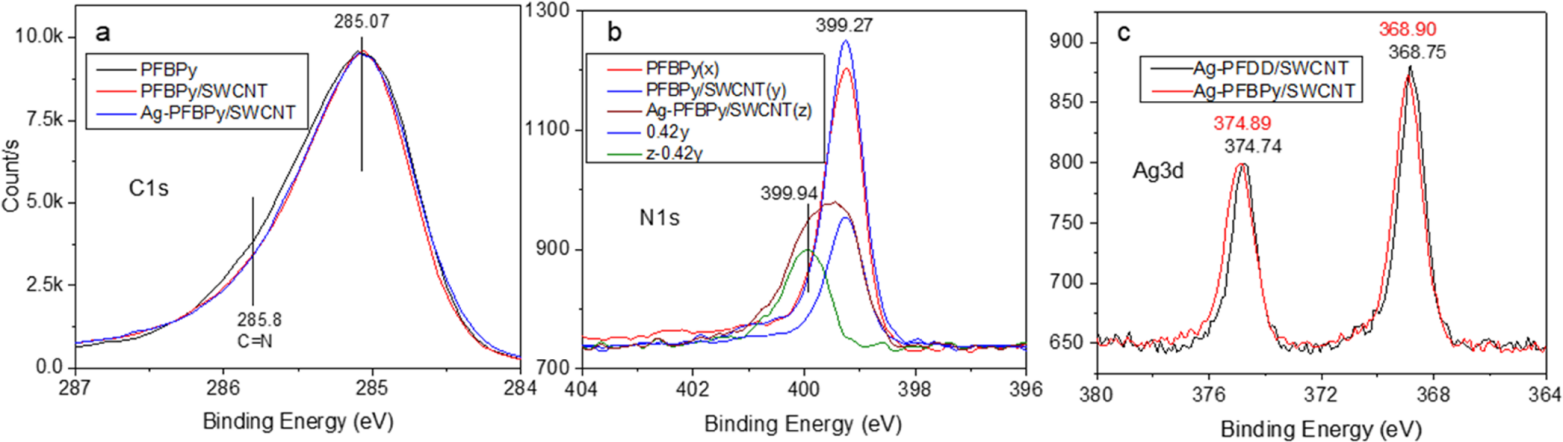
High-resolution XPS spectra of the series of PFBPy and PFDD samples. (a) C 1s curves of the PFBPy samples. It shows that both PFBPy/SWCNT and Ag–PFBPy/SWCNT have a narrower peak than the pure PFBPy. (b) N 1s curves of PFBPy (*x*), PFBPy/SWCNT (*y*), and Ag–PFBPy/SWCNT (*z*). The broad peak *z* can be deconvoluted into two components (0.42*y*), and the resulting peak (*z* – 0.42*y*) corresponds to Ag-coordinated PFBPy and free PFBPy with an area ratio of 0.48/0.52. (c) Ag 3d curves of the two Ag-doped samples. It indicates a 0.1–0.2 eV shift to higher energies with a 0.1 eV increase in FWHM (from 1.11 to 1.21 eV) of the PFBPy sample, consistent with the smaller size of the AgNPs in this sample.^49^

XPS was also used to study the interaction of the components in these two series of samples at the pure polymer, polymer/SWCNT, and Ag–polymer/SWCNT composite phases, where the Ag–OTf-doped samples have a [Ag]/[CNT] ratio of 0.0183 (0.4 [Ag]/[BPy] for PFBPy composite). The XPS survey scan (Figure S4) confirmed the presence of N in the three PFBPy samples and Ag and F in the Ag–OTf doped composites. The high-resolution C 1s curves depicted in Figure S5c indicate a pyridine structure in PFBPy.^15,44^ Additionally, Figure 6a shows that the C 1s peak in the PFBPy/SWCNT and Ag–PFBPy/SWCNT composites is narrower than that in the pure polymer, with the full width at half-maximum (FWHM) decreasing from 1.01 to 0.92 eV. This finding suggests that the nitrogen of PFBPy on the SWCNTs has a more uniform chemical environment unaffected by Ag coordination. This alternation can be explained by the conformational change of the polymer as soon as it wraps around the nanotubes. It is well known that a BPy unit in its free state can take both cis- and trans-conformations. Computer modeling indicated that the trans-conformation is more stable than the cis-conformation in solution owing to its lower steric hindrance.^45^ However, the trans-conformation will convert to cis- on some solid surfaces, such as on a silver surface, even when the surface is covered with a self-assembled monolayer.^46^ A positive charge on this type of surface is required for this trans-to-cis conversion, as the positively charged surface attracts the nitrogen lone pair electrons on the BPy to reorient the BPy unit to the cis-conformation.^46^ Notably, SWCNTs are typically positively charged by the O_2_/H_2_O redox pair in air,^39^ and thus, doped nanotubes are able to induce this trans- to cis- conversion as soon as the polymer approaches the nanotube surface. This process will facilitate the Ag coordination reaction when Ag–OTf is added to a PFBPy/SWCNT solution.

In Figure 6b, the N 1s peaks of the three PFBPy materials are compared. It shows that little change occurred after the polymer was combined with SWCNTs. After Ag–OTf was added into the composite, the spectrum broadened significantly on the side with higher binding energy. Two components effectively resolved this peak at 399.27 and 399.94 eV, corresponding to the free BPy and Ag-coordinated BPy in the polymer, respectively.^47,48^ Notably, the interaction of the pyridine unit with the metal NPs and metal ions has the same effect on the N 1s spectrum due to the presence of an oxide layer on the surface of metal NPs.^47^ In the presence of air, the formed AgNPs will be slowly oxidized to form an oxide layer on the surface; meanwhile, the in-situ photoreduction under room light will reduce the oxide to metallic silver, and finally, an equilibrium will be reached with a thin layer of Ag_2_O formed on the surface. This effect will efficiently preserve the coordinating interaction of AgNPs with PFBPy. Combining the π–π interaction of the polymer with nanotubes, this effect will ensure a tight anchoring of the AgNPs to the SWCNT surfaces and enable good electrical contact to promote charge transfer. This interaction was also confirmed by the Ag 3d spectrum of the Ag–OTf-added samples, as shown in Figure 6c. The Ag 3d peaks of the Ag–PFBPy/SWCNT sample shift by 0.1∼0.2 eV to higher binding energies with a 0.1 eV increase in FWHM compared to the Ag–PFDD/SWCNT sample,^49^ meaning a broader electronic state of silver in the PFBPy sample, associated with the presence of BPy chelated silver and metallic silver. This result is also consistent with the TEM study in the following section, showing that the AgNP size in the PFBPy sample was significantly smaller. A previous study reported an increase in the binding energy with AgNPs smaller than 4 nm.^38,50^ A similar phenomenon was also observed for another noble metal, Pd.^50^

### 3.5 TEM Investigation

**Figure 7:**
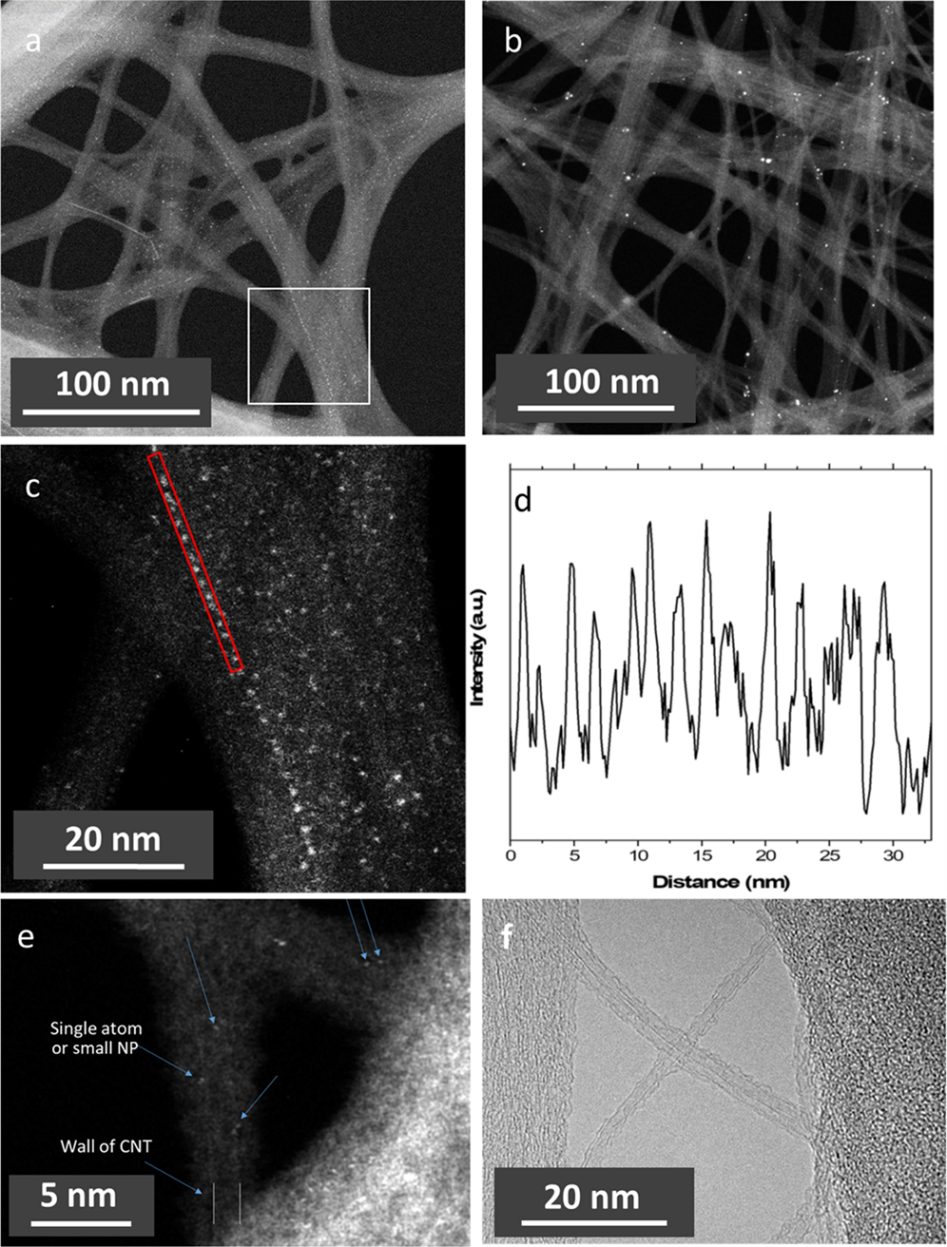
Annular dark-field (ADF) and high-resolution transmission electron microscopy (HRTEM) images of the Ag–PFBPy/SWCNT and Ag–PFDD/SWCNT composites deposited on a lacey carbon-film-coated TEM grid: ADF-STEM image of (a) the Ag–PFBPy/SWCNT composite and (b) the Ag–PFDD/SWCNT composite, where a high-angle annular dark-field Fischione detector in scanning transmission electron microscopy (STEM) mode was used. (c) An enlarged image of the selected area in (a) (white square) clearly shows a AgNP chain. (d) A density graph of the AgNP chain in the red strip in (c). (e) The high-magnification image of Ag–PFBPy/SWCNT at a thinner area using a low-pass filter to show the nanotube walls and very tiny AgNPs on the nanotube. (f) The HRTEM image of the Ag–PFBPy/SWCNT composite showing the wall of nanotubes and the wrapping polymer.

HRTEM and ADF images of the Ag–PFDD/SWCNT and Ag–PFBPy/SWCNT composites were captured on lacey carbon-film-coated copper grids. To obtain sufficient contrast for nanotubes with a diameter of ∼1.3 nm, only the images from the holes of the carbon-supporting film were captured. Under the influence of a high-energy electron beam, only aggregated nanotubes could be stably suspended to get a clear image due to the lack of support in the imaging area. Figure 7a,b compares the dark-field (ADF-STEM) images of the Ag–PFBPy/SWCNT (a) and Ag–PFDD/SWCNT (b) composites at comparable magnification. It shows that the Ag–PFBPy/SWCNT sample contains a large number of tiny white dots smaller than 1 nm, whereas the Ag–PFDD/SWCNT (Figure 7b) sample contains much larger white dots ranging in size from 1 to 5 nm but a small population. These white dots are attributed to AgNPs. The AgNPs in Figure 7a are hard to see, and thus an image at a larger magnification was captured and displayed in Figure 7c. Figure 7a,c shows an NP chain consisting of more than 60 particles. These NPs uniformly aligned along the nanotube aggregate. The graphical analysis of the chain (Figure 7d) in the red strip in Figure 7c reveals an average particle size of 0.6 nm and the typical minimum separation between adjacent NPs of ∼1.7 nm. It is consistent with the distance between the two adjacent BPy units (1.7 nm) in the polymer chain. This result suggests that the BPy unit of the wrapped PFBPy polymer seeded and anchored the AgNPs on the nanotube surface. However, the distance between the adjacent NPs in the chain is not uniform, probably due to the preferential growth of NPs on certain crystal planes of AgNPs. These types of AgNP chains with varying lengths were often observed in the sample. The image of a very thin area of the Ag–PFBPy/SWCNT sample (Figure 7e) reveals the existence of significantly smaller particles with a size close to a single atom. In this image, the wall of a carbon nanotube can also be identified. Figure 7f displays a conventional HRTEM image of the Ag–PFBPy/SWCNT composite for nanotube images, in which a single nanotube and a two-nanotube bundle are seen between larger bundles. In this image, no AgNPs are visible due to the low contrast and small particle size. This image clearly displays a polymer layer on the nanotube surface, demonstrating that the SWCNTs are well wrapped with the polymer.

Because the sample was prepared by adding a Ag–OTf solution to a PFBPy/SWCNT solution in THF under an ambient lighting condition, these bright particles are attributed to photoreduced AgNPs. This type of Ag reduction by light is commonly observed in Ag–BPy supramolecular structures.^51^ Notably, the Ag–OTf solution was added to the composite solution by gently applying droplets without stirring to allow the Ag–OTf molecules to diffuse slowly into the solution. We believe that the larger-sized AgNPs may form in an area with high Ag^+^ concentrations near the Ag–OTf droplets. Under light irradiation, the BPy chelated Ag^+^ was reduced to Ag^0^ by the electrons injected from the SWCNT. This process also released the BPy unit, allowing it to capture subsequent Ag^+^ at this position to grow the AgNPs. As a result, AgNPs can be formed on the SWCNT surfaces, with their location fixed by the BPy units of the wrapped PFBPy. On the other hand, As soon as Ag^+^ ions are coordinated with BPy units, their mobility will be limited, resulting in a slower accumulation of Ag^+^ ions on the formed NPs; in addition, with the low Ag^+^ usage (0.4 [Ag]/[BPy]), this factor will restrict the formed NPs to a small size. It also indicates that Ag^+^ coordination with BPy is a faster process than Ag^+^ reduction. This benefited from the cis-conformation of the BPy unit on the nanotube, as confirmed by the XPS study. Additionally, the high-magnification image of a thinner area in Figure 7f obtained by using a low-pass filter confirmed the presence of very small AgNPs with sizes close to a single atom. In contrast, large AgNPs (1–5 nm) were detected in the Ag–PFDD/SWCNT composite, as shown in Figure 7b, because the AgNPs in this sample were formed by simple diffusion-controlled Ag^+^ reduction under ambient lighting conditions, where as soon as a Ag^0^ seed forms on the SWCNT surface, it generates an interface with SWCNT to promote the exciton separation, leading to further Ag^+^ reduction and the growth of larger AgNPs.

In the PFBPy sample, the efficient coordination of BPy with Ag cations not only tightly anchors the formed AgNPs on the SWCNT surface but also regulates the concentration of free Ag^+^ and thus effectively controls the AgNP size at the sub-nm level. This effect can be further confirmed by the result shown in Figure S7, where the ADF images of three Ag–PFBPy/SWCNT samples with the Ag^+^ usage ([Ag]/[BPy]) increased from 0.4 to 5.0 and further to 50 were compared. It indicates that the average size of AgNPs is about 0.3, 0.6, and 0.8 nm, respectively. This result confirmed that the size of the formed AgNPs in the PFBPy/SWCNT solution could be easily controlled at the sub-nm level over a wide [Ag]/[BPy] range, with the small-sized NPs being stabilized by the combined effect of PFBPy anchoring and SWCNT-sensitized Ag^+^ photoreduction in the Ag–PFBPy/SWCNT sample. However, this effect did not occur in the Ag–PFDD/SWCNT sample due to the absence of the anchoring effect. This feature is crucial for the preparation of small, highly dispersed metal NP composites, especially considering that relatively large size particles were prepared in the common-used techniques, such as in the self-assembled NP composites.^51^

### 3.6 TFT Study

**Figure 8:**
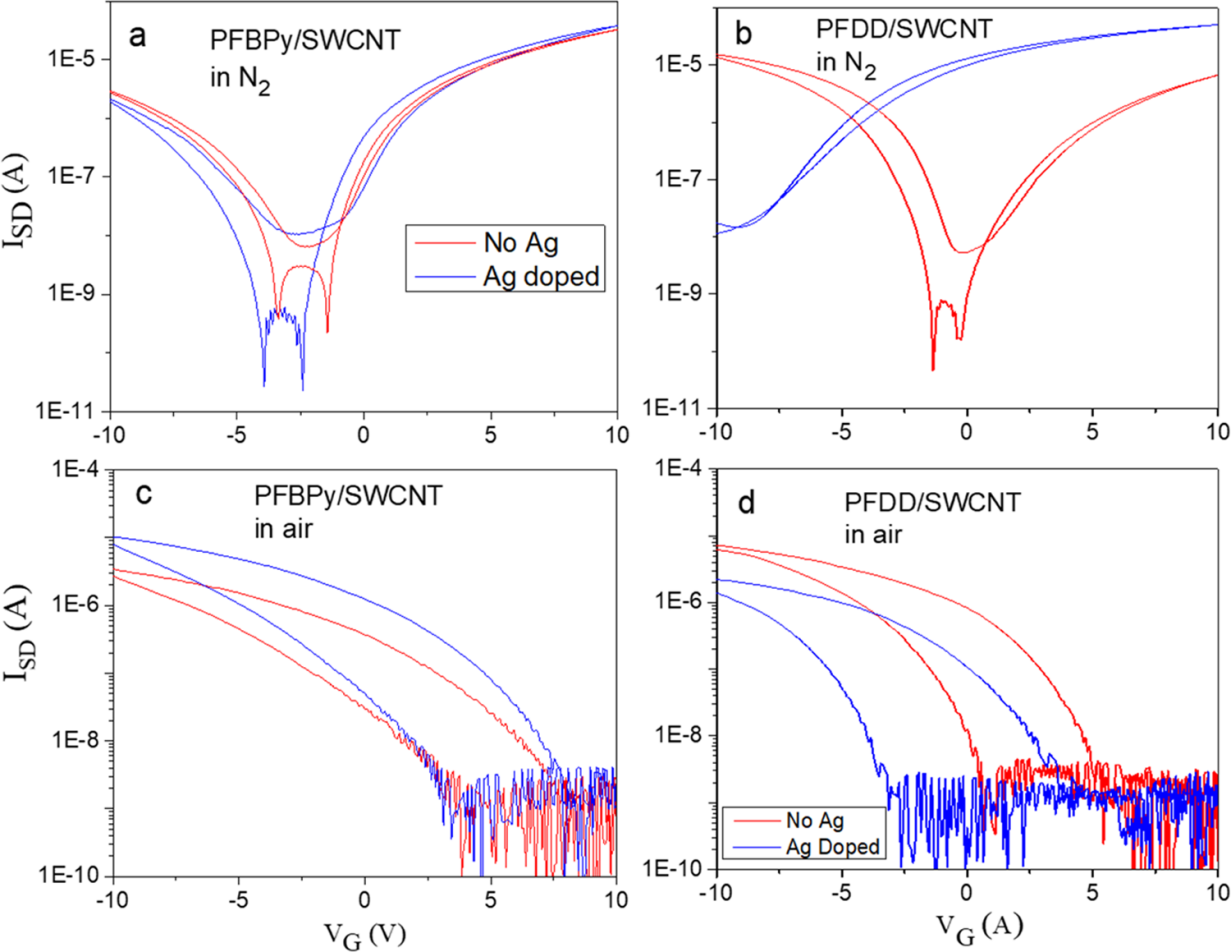
Transfer curves of the TFT devices with a 20 μm channel length of PFBPy/SWCNT (a, c); PFDD/BPy (b, d), with and without AgNPs. The AgNP composites were prepared by adding 0.4 equiv of Ag–OTf into the corresponding composite solution in THF and then coated to the device by drop casting. The device was annealed at 200 °C in a nitrogen glovebox overnight, and the transfer curves were recorded in N_2_ (a, b). Afterward, the devices were placed in ambient air for 1 h, and the transfer curves were collected in air (c and d).

The TFT devices of the PFBPy/SWCNT and PFDD/SWCNT samples, as well as their Ag–OTf doped samples at [Ag]/[BPy] = 0.4 or [Ag]/[CNT] = 0.018, were evaluated in N_2_ and air. To minimize the doping effect of adsorbed moisture and oxygen on TFT devices, Fraunhofer chips containing 4 × 4 devices with channel lengths of 2.5, 5.0, 10.0, and 20.0 μm and a channel width of 1 mm were coated with the corresponding materials and annealed overnight at 200 °C in a N_2_ glovebox. Figure 8a,b shows the comparison of their transfer curves. A characteristic SWCNT ambipolar character was observed in the PFBPy/SWCNT and PFDD/SWCNT composites, where n-branch and p-branch appeared, according to the previously reported findings.^52^ A relatively stronger n-branch of the PFBPy/SWCNT device indicated that the p-doping level of the SWCNTs was reduced due to the presence of the PFBPy polymer on the surface, where the lone pair of electrons from the pyridine nitrogen partially donated charge to SWCNTs, as observed in the XPS study.

Ag–OTf doping had a very distinct effect on the TFT performance of the PFDD/SWCNT and PFBPy/SWCNT devices, as shown in Figure 8. It changed the PFDD/SWCNT device from an ambipolar to an n-type, but the PFBPy/SWCNT device remained unchanged. This variation is attributed to the different sizes of AgNPs formed in the samples. According to the TEM study, 1–5-nm-sized AgNPs predominated in the Ag–PFDD/SWCNT composite. Due to the lower work function (4.48 eV) of the AgNPs attributed to the large particle size,^53,54^ the Fermi level equilibrium between the AgNPs and SWCNTs caused electron flow into the SWCNTs to convert this material into an n-type semiconductor (see Figure S8). However, the Ag–OTf doping of the PFBPy/SWCNT composite did not induce an apparent change in the transfer curve, indicating that the Fermi level alignment did not induce an apparent doping level change in the SWCNTs, meaning that the AgNPs in this material had the same Fermi level as the SWCNTs, most likely due to the extremely small size of the formed AgNPs, ranging from a few atoms to 1 nm. According to a prior study, the work function of AgNPs exhibits a significant size dependence.^38^ Plieth’s equation shows that the increase in the work function of NPs relative to the bulk metal is inversely proportional to the particle radius,^55^ and therefore, the size effect is most prominent at sub-nm levels.

Consequently, it is anticipated that the smaller AgNPs in the PFBPy composite will exhibit a significant increase in their work function. It resulted in a matched energy level and a reduced electron flow to the SWCNTs (see Figure S8). This feature significantly increased the sensing sensitivity of the Ag–PFBPy/SWCNT composite material, as shown in Figure 1, which exhibits a 5-fold increase in sensing response to a small variation in air humidity compared to the controlled device. At the aligned energy level of NPs with SWCNTs, any small variation in the NP energy level induced by the surface adsorption would result in an apparent doping level change in the SWCNT and generate an excellent sensing response.

Transfer curves of PFBPy/SWCNT and PFDD/SWCNT composites with and without Ag doping in the air (Figure 8c,d) were notably distinct from those in N_2_. All of the materials were changed to p-type, indicating that the p-doping effect of the H_2_O/O_2_ redox pair outweighed the n-doping effect of the AgNPs. This effect is frequently observed in SWCNT materials, where the pH value of the nanotube surface controls the doping level.^39^ However, the aligning effect of the Fermi level may still be observed, as illustrated in Figure S8c. Under these circumstances, the AgNP electron-donating effect may still be observed in the PFDD/SWCNT composite, where the Ag-doped sample exhibited a lower p-branch than the nondoped sample. But PFBPy/SWCNT behaved differently. The Ag-doped sample has a larger p-branch than the nondoped sample. This is also attributed to the higher Fermi energy of AgNPs in the Ag–PFBPy/SWCNT composite due to their smaller sizes (Figure S8c).

It has been reported that the work function of AgNPs is considerably affected by ligands adsorbed on their surfaces.^39,53,54^ Due to the large dipolar moment of the formed complex structures, when the surface of the AgNPs is completely coated with a highly polar ligand, the work function is projected to increase by up to 0.7 eV.^53,54^ This effect can significantly increase the electron flow from the SWCNTs into AgNPs as the ligand concentration increases. In our composite materials, it is important to note that the AgNPs were formed by photoreduction in the Ag–OTf /polymer/SWCNT composite solutions, and a portion of the released ^–^OTf ligands were adsorbed onto the AgNP surfaces, increasing the work function of the AgNPs. This interaction is advantageous for moisture sensing. As a dry air pulse was applied during the sensing process, RH was reduced to a low value. It resulted in a decrease in the adsorbed water layer on the AgNP surface, leading to an increase in the concentration of the ^–^OTf ligand, followed by an increase in the Fermi level of the AgNPs, and finally an increase in electron flow from SWCNTs, leaving a high hole concentration there. As a result, a large Δ*G*/*G*_0_ was detected, as shown in Figure 1.

Therefore, the change in the adsorbed moieties on the AgNP surface is crucial to this sensing enhancement in the Ag–PFBPy/SWCNT composite material. Additionally, AgNPs in the composite have a more hydrophilic surface than the other components, PFBPy and SWCNT, since they are covered with a thin oxide layer coupled with superhydrophilic ^-^OTf ligands.^56^ This layer enhances the Fermi level change by preferentially adsorbing polar molecules on the surface. The close contact of the AgNPs with the SWCNTs will therefore allow the signal to be readily transmitted to the SWCNTs. Therefore, the significant improvement in the AgNP sensor over a non-AgNP sensor can be attributed to the following factors: (1) The relatively hydrophilic surface of the AgNPs ensures the selective adsorption of the target molecules and amplifies the effect of the concentration change of the adsorbed moieties. (2) Due to the small size of the AgNPs, their Fermi level is aligned with that of the SWCNTs, allowing for sensitive charge transfer between them. (3) The tight anchoring of the AgNPs to the SWCNT surfaces enabled by the wrapped PFBPy polymer ensures good electrical contact to promote charge transfer. This anchoring effect is secured by the AgNPs chelated with the BPy units in the wrapping polymer due to the presence of an oxide layer on the NPs and strong π–π interactions between the polymer main chain and the nanotube. These factors facilitate a charge transfer to deliver the sensing signal from the AgNPs to the nanotube to increase the sensing sensitivity and durability as well.

## 4 Conclusions

We have demonstrated the existence of efficient transduction in Ag–PFBPy/SWCNT composites for humidity sensors. In this study, two series of PFBPy and PFDD samples (with or without alternating BPy units in the conjugated polyfluorene chain) were synthesized and studied at three compositing stages, i.e., pure polymer, polymer/SWCNT, and Ag–polymer/SWCNT. The AgNPs were introduced into the polymer/SWCNT composites by applying a Ag–OTf solution to the polymer/SWCNT composite solution at a [Ag]/[CNT] molar ratio of 0.0183, where Ag^+^ was first chelated with BPy and then reduced to Ag^0^ on the nanotube surface catalyzed by room light. The formed Ag^0^ atoms served as seeds for the subsequent Ag^+^ deposition and reduction to form AgNPs. Due to BPy chelation, the diffusion of Ag^+^ cations was restricted, and as a result, the size of the AgNPs was limited to ∼0.3 nm. This process was evidenced by the formation of AgNP chains along PFBPy polymer backbones, as observed by HRTEM. This particle size aligned the Fermi energy of the AgNPs with that of the SWCNTs to facilitate the efficient charge transfer between them during sensing. However, in the absence of BPy chelating in the PFDD/SWCNT solution, the reduction of Ag^+^ is diffusion-controlled, and the formed AgNPs are much larger with an upward shift in their Fermi level and with a relatively loose contact with SWCNTs. Sensing devices fabricated from the Ag–PFBPy/SWCNT composite demonstrated a high sensitivity to humidity detection with a 5-fold higher response than the control device without AgNPs. The humidity effect was amplified by the selective adsorption of moisture on the AgNP surfaces to generate an energy level change. This signal is easily transferred to the carbon nanotubes owing to the excellent contact between AgNPs and SWCNTs, enabled by the chelating interactions of Ag with the BPy unit in the polymer and the π–π interactions of the polymer backbone with the nanotubes. Furthermore, BPy can be used as a ligand for other metals; hence, this compositing system is also ideal for anchoring other metals that would target distinct analyte detection and also catalysis.

## Supporting Information Available

(1) Sensing setup; (2) kinetic discussion of the Ag^+^ reduction in the polymer/SWCNT composite solutions in THF; (3) absorption spectroscopy study at solid states; (4) H–OTf doping into the PFBPy/SWCNT composite solutions in THF; (5) XPS spectra of the PFBPy and PFDD film samples at three different compositing stages; (6) HRTEM analysis of the Ag–PFBPy/SWCNT composite; and (7) improvement of humidity sensing capability of the AgNP-doped PFBPy/SWCNT composite (PDF)
