# Supplementary material for: Silver Nanoparticles Anchored on Single-Walled Carbon Nanotubes via a Conjugated Polymer for Enhanced Sensing Applications
Source: ACS Omega. 2023 Apr 5;8(15):14219–32. doi: 10.1021/acsomega.3c01127 (PMC10116552; doi:10.1021/acsomega.3c01127)
Supplement: Supplementary file 1 — ao3c01127_si_001.pdf [file ao3c01127_si_001.pdf]

Supporting Information:

Silver Nanoparticles Anchored on Single-walled Carbon Nanotubes via a Conjugated Polymer for Enhanced Sensing Applications

Jianfu Ding*[a], Zhao Li[a], Oltion Kodra[b], Martin Couillard[b] Jianying Ouyang[a], François Lapointe[c], Patrick R. L. Malenfant*[c]

[a] Security and Disruptive Technologies Research Centre, National Research Council of Canada, 1200 Montreal Road, M-12, Ottawa, ON, Canada, K1A 0R6

[b] Energy, Mining and Environment Research Centre, National Research Council of Canada, 1200 Montreal Road, M-12, Ottawa, ON, Canada, K1A 0R6

[c] Security and Disruptive Technologies Research Centre, National Research Council of Canada, 1200 Montreal Road, M-50, Ottawa, ON, Canada, K1A 0R6

Corresponding Authors:

Jianfu Ding, e-mail: jianfu.ding@nrc-cnrc.gc.ca.

Patrick R. L. Malenfant, e-mail: patrick.malenfant@nrc-cnrc.gc.ca.

1. Sensing setup

The setup used for the humidity sensor test is illustrated in Figure S1.

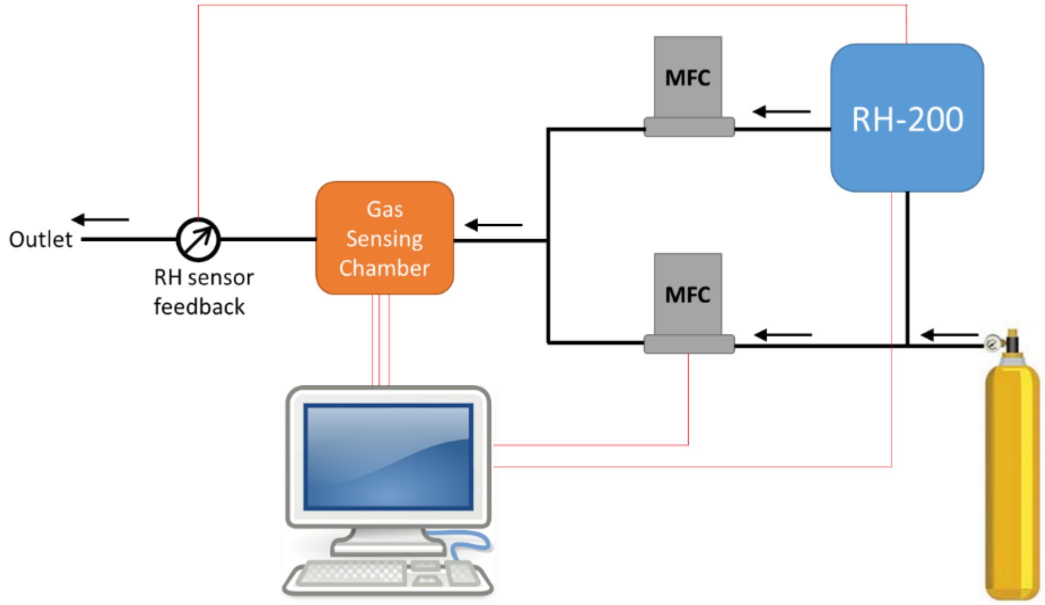

Figure S1, Sensing setup for humidity testing. Dry air from the cylinder was connected to an RH-200 Relative Humidity Generator (L&C Science and Technology) for feeding air of 50% RH as the carrier gas to the sensing chamber at a constant flow rate of 250 sccm controlled by a mass flow controller. A sequence of 10 s dry air pulses were introduced into the sensing chamber via an MKS MFC P4B (range 500 sccm) at flow rates of 10, 20, 30, 40 and 50 sccm, followed by a 60 s pause, corresponding to the corrected RH levels in the testing chamber of 48.1%, 46.3%, 44.6%, 43.1%, and 41.7%.

2. Kinetic discussion of the Ag^+ reduction in the polymer/SWCNT composites solutions in THF

Figure 2b indicates a significant decrease in the intensity of the S11 and S22 peak of the PFBPy/SWCNTs solution in the presence of 0.4-equivalent Ag–OTf (0.0183 [Ag]/[CNT])) as it is exposed to light from a Xenon lamp at an intensity of 110 W/m^2. This light is ~1000 times stronger than the intensity of the dim room light (0.11 W/m^2). Thus, it was believed that photo-catalyzed Ag reduction occurred in the solution. To study the effect of the PFBPy to this reaction, the reaction in PFBPy/SWCNT was compared with those in PFDD+BPy/SWCNT and PFDD/SWCNT (2.5/1, w/w) solutions with Ag–OTf at 0.0183 [Ag]/[CNT] under the same condition, with the results displayed in Figure 3b and c, respectively.

For this Ag^+ reduction on the SWCNT surface, excitons were generated in SWCNTs and the electrons were extracted to Ag^+ to convert it to Ag^0. Thus the reaction is dependent on the efficient contact with the SWCNTs for charge transfer, and thus, the reaction can be simplified as follows:

$$Ag^+ + CNT^* \implies Ag^0\text{-}CNT \quad (1)$$

where CNT* is the effective contact site on a SWCNT to transfer electrons to Ag^+. Therefore, the speed equation can be expressed as:

$$\frac{d[Ag]}{dt} = -k[Ag][CNT^*] \quad (2)$$

Integrating this equation by using $[Ag] = [Ag]_0$ at t=0 results in the following equation:

$$\frac{[Ag]}{[Ag]_0} = e^{-k[CNT^*]t} \quad (3)$$

Based on Eq. 1, $[Ag]/[Ag]_0$ can be represented by $(1-[Ag^0]/[Ag]_0)$, where Ag^0 is the reduced Ag; thus, Eq. 3 can be rewritten as:

$$\frac{[Ag^0]}{[Ag]_0} = 1 - e^{-k[CNT^*]t} \quad (4)$$

This equation can be re-written by introducing [CNT], the molar concentration of SWCNT to the both sides as a concentration reference to yield:

$$\frac{[Ag^0]}{[CNT]} = \frac{[Ag]_0}{[CNT]}(1 - e^{-k[CNT^*]t}) \quad (5)$$

For kinetic analysis, a correlation between the change in $(S11)/(S11)_0$ and $[Ag^0]/[CNT]$ must be measured. Therefore, the reaction shown in Figure 3a was examined under stronger light by using a 610 W/m^2 Xenon lamp to assure the complete conversion of Ag^+ during the reaction time (8 min). The absorption spectrum of the reaction solution became unchanged at this time, indicating that all Ag^+ was converted. Thus, this PFBPy/SWCNT solution was supplemented with 0.011, 0022, 0.033, 0.044 and 0.055 equivalents (over SWCNT) of Ag–OTf and irradiated for 8 min under the Xenon lamp. Figure 3d depicts the absorption spectrum of the solution at each step. The plot of $(S11)/(S11)_0$ vs. $[Ag^0]/[CNT]$ is displayed in Figure 3e, which resulted in a fitting curve (Eq 6).

$$\frac{(S11)}{(S11)_0} = 0.50(1 + e^{-\frac{[Ag^0]/[CNT]}{0.024}}) \quad (6)$$

This equation was then used as the calibration for the Ag^+ conversion calculation in the reaction depicted in Figure 3a, b and c, where $[Ag]_0/[CNT]$ at various reaction times can be calculated from the value of $(S11)/(S11)_0$. The calculated results for the reactions with PFBPy/SWCNT, PFDD + BPy/SWCNT and PFDD/SWCNT are plotted in Figure 3f. Fitting the data with Eq. 5 produced excellent fitting curves for these three reactions, yielded identical values for the first constant of $[Ag]_0/[CNT] = 0.025$ and the second constant of $k[CNT^*] = 0.111$, 0.0105 and 0.00189. The first constant is slightly greater than the feed ratio of $[Ag]_0/[CNT] = 0.0183$. The second constant has two components, the speed constant k and the effective SWCNT concentration ([CNT*]) for charge transfer. Because k is a constant for the three reactions depicted in this figure, the difference in this value can result in $[CNT^*]_{PFBPy}/ [CNT^*]_{PFDD+BPy} /[CNT^*]_{PFDD} = 0.111/0.0105/0.00189 = 58.7/5.6/1.0$, indicating that the number of effective charge transfer sites is ~59 times greater in

the PFBPy/SWCNT solution than in the PFDD/SWCNT solution, while it only increased only 5.6 times upon the addition of 1-equivalent 2,2-BPy ([BPy]/[FDD]=1/1) to the PFDD/SWCNT solution. In this PFDD + BPy/SWCNT solution, the formed Ag–BPy complex could also be adsorbed onto the SWCNT surfaces due to the π-π interactions of BPy with the SWCNTs to facilitate the Ag^+ anchoring effect, however, its strength was much lower than that of the PFBPy copolymer due to the extremely small π structure of the BPy molecule. This result confirmed the large coplanar PFBPy polymer main chain has significant effect on anchoring silver on the SWCNT surface to enable efficient in situ Ag^+ photoreduction.

3. Absorption spectroscopy study at solid states

In a TFT or sensor device, the active material, SWCNTs, is a thin solid layer; hence, the effect of Ag–OTf doping was also investigated on solid samples. The polymer/SWCNT composite solutions and their Ag–OTf-doped solutions were coated onto a quartz slide to form films. Their absorption spectra were collected and compared in Figure S2 for the PFBPy/SWCNT (a) and PFDD/SWCNT composites (b). The spectra reveal that the S11 and S22 peaks of the Ag–OTf-doped film samples substantially broadened, and their intensities decreased to less than half of what they were before Ag–OTf addition. This result revealed that the SWCNTs in the Ag–PFBPy/SWCNT and Ag–PFDD/SWCNT solid samples were heavily doped. Interestingly, Figures S2a and S2b also demonstrate that the S11 and S22 peaks were partially restored after the films were soaked in ethanol for 10 min, with intensities reaching ~70% of those of the undoped samples. This suggests that EtOH soaking decreased the amount of SWCNTs doping, indicating that some doping reagents were removed during soaking. During the sample preparation, the added Ag–OTf was photoreduced to Ag^0, this reaction generated a hole in the SWCNT and release a $^{-}$OTf anion that will adsorb on the SWCNTs to balance the hole. The H_2O/O_2 redox gradually neutralizes the excess hole in SWCNTs and generates a proton on the SWCNT surface upon contact with air. It converted $^{-}$OTf to H–OTf. Due to the extreme acidity of this molecule, it will be easily removed during EtOH washing, leaving largely dedoped SWCNTs with restored S11 and S22 peaks.[1]

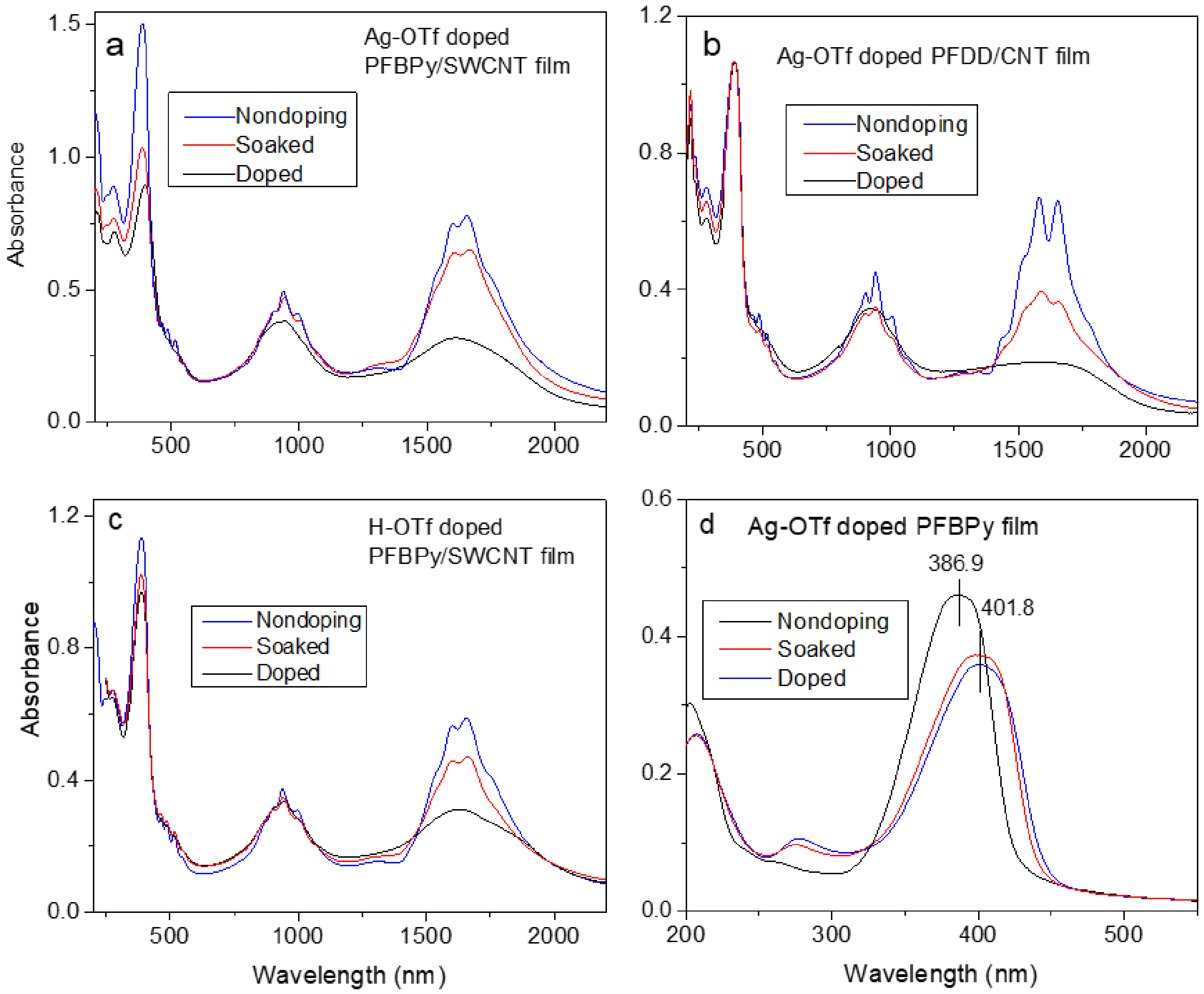

Figure S2. Variation in the absorption spectra of polymer/SWCNT composite films upon doping with Ag–OTf and de-doping by EtOH soaking: (a) PFBPy/SWCNT; (b) PFDD/SWCNT. The doping effect of the corresponding acid, H–OTf, on the PFBPy/SWCNT film is also compared in (c). In this test, the PFBPy/SWCNT or PFDD/SWCNT composite solutions at a polymer/SWCNT weight ratio of 2.5/1 were added with Ag–OTf or H–OTf solution at 0.0183 [Ag]/[CNT] or [H]/[CNT] ratio, corresponding to 0.4 equivalent Ag^+ or H^+ to BPy. The films were prepared by coating the solution on quartz plates and annealing at ~ 50 °C for 10 min. Then, they were de-

doped by soaking in EtOH for 10 min and dried at 150 °C for 10 min. For comparison, the doping and soaking effects on the pure PFBPy film without the presence of SWCNTs is displayed in (d).

This hypothesis was validated by an H–OTf doping experiment as shown in Figure S2c, in which the PFBPy/SWCNT film was doped with H–OTf and subsequently washed with EtOH. The addition of 0.4-equivalent H–OTf produced a similar change in intensity as that of Ag–OTf doping. This interaction is also reflected by the variation of the polymer absorption peak.[2] As shown in Figure S2a, the addition of Ag–OTf broadened and shifted the PFBPy peak from 387.0 to 397.5 nm, showing an Ag^{+} coordinating with the BPy unit, whereas the peak returned to 387.0 nm following EtOH washing due to the removal of H–OTf after Ag^{+} was photoreduced to Ag^{0} by light catalysis. However Figure S2d demonstrates that EtOH soaking had no apparent effect on the Ag–OTf-doped PFBPy film devoid of SWCNT, although the Ag–OTf doping also triggered polymer peak broadening and redshift due to the formation of an Ag complex with BPy, indicating this structure remained at the complex state and thus no $^{-}$OTf was removed during EtOH soaking. This result revealed that SWCNT is a crucial component for converting Ag^{+} to Ag^{0}.

4. H–OTf doping into the PFBPy/SWCNT composite solutions in THF

As shown in Figure S2, removing H–OTf from the composite film by EtOH soaking significantly reduced the SWCNT doping level, with the S11 peak recovering partially. This feature indicates that H–OTf is a strong SWCNT doping agent. To verify this, Figure S3 illustrates the variations in the absorption spectra of a PFBPy/SWCNT solution during H–OTf titration. Due to the doping effect and protonation of the BPy unit, H–OTf titration significantly reduced the S11 peak and broadened the polymer peaks. However, this titration does not result in the redshift of the S11 and S22 peaks, as seen in Figure 2a for the Ag–OTf titration, indicating that the redshift is attributable to dielectric screening effect associated with the formation of Ag–BPy complex in the PFBPy wrapped on the SWCNT surface.

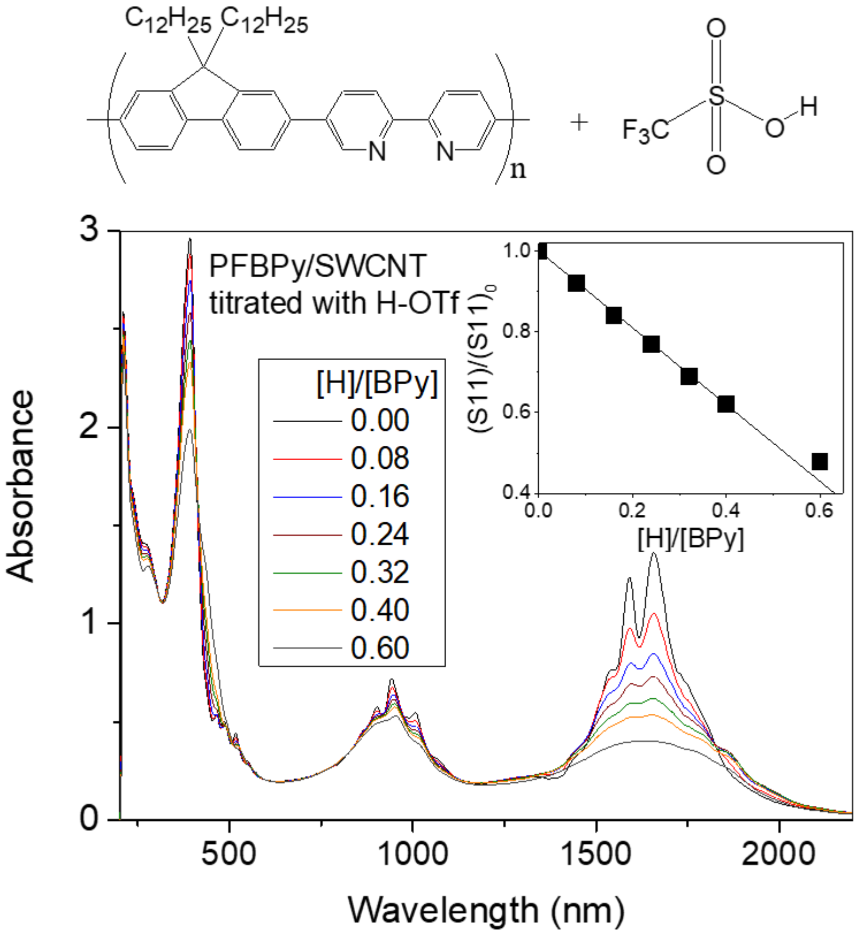

Figure S3. Variation of the UV spectrum of the PFBPy/SWCNT composite solution at a Polymer/SWCNT weight ratio of 2.5/1 in THF with the addition of H–OTf at [H]/[BPy] varying from 0.00 to 0.08. 0.16, 0.24, 0.32, 0.40, and 0.60. The change in the S11 peak intensity with [H]/[BPy] is plotted in the inset.

5. XPS spectra of the PFBPy and PFDD film samples at three different compositing stages

Figure S4 compares the survey spectra of the PFDD and PFBPy series samples. On the PFDD and PFBPy polymer curves, a weak O 1s peak accompanied by Si peaks was seen. They are attributed to the silica gel contamination introduced during the column chromatography purification in polymer synthesis. The disappearance of the Si peak, and the weakening of the O 1s peak in the composite materials indicate that this contamination was removed by centrifugation

during the composite sample preparation. An N 1s peak in all three PFBPy samples was attributed to pyridine N. After Ag–OTf was added to the polymer/SWCNT composite solutions, a double Ag 3d peak with a weak F 1s peak were seen, confirming the presence of OTf species.

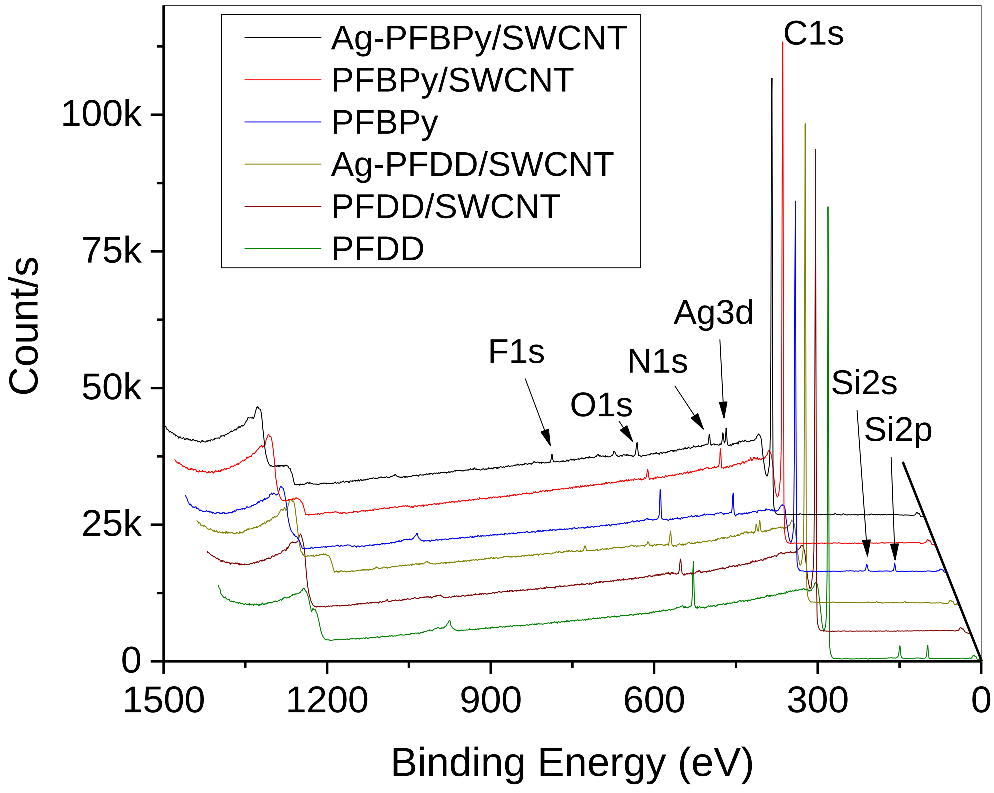

Figure S4. Comparison of the XPS survey spectra of the six films of the PFBPy and PFDD samples at three different compositing stages, i.e., pure polymer, polymer/SWCNT composites and Ag-polymer/SWCNT composites. A 0.0183 [Ag]/[CNT] molar ratio was used in the Ag-doped film samples (0.4 [Ag]/[BPy] for the Ag–PFBPy/SWCNT composite). The films were prepared by coating the corresponding solutions on an aluminium plate.

Figure S5 compares the high-resolution C 1s curves of the six samples. For PFDD and its SWCNT, and Ag/SWCNT composites, Figure S5a displayed no apparent change after the polymer was composited with the SWCNTs and even after it was doped with Ag–OTf. However, Figure

S5b depicted that C 1s curves of PFBPy became narrower in the SWCNT and Ag/SWCNT composites. This is attributed to that all the BPy units in the polymer have cis-conformation in the composite sample, while in the pure polymer, both cis- and trans-conformation are adopted. When comparing the PFDD and PFBPy pure polymers (Figure S5c), the C 1s peak broadened to a high binding energy for PFBPy. This peak was resolved by subtracting the PFDD peak at 285.0 eV to produce a new peak at 285.8 eV (red curve), which was assigned to aromatic carbons in the pyridine unit,[3-5] with a relative intensity of ~10%, consistent with the –C=N content in this polymer.

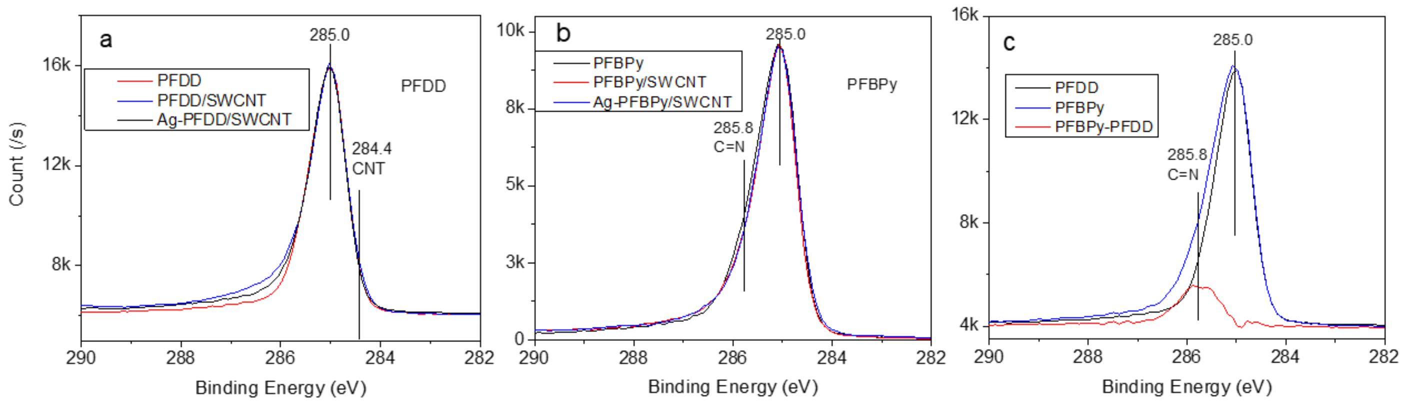

Figure S5. C 1s band of the polymers, their SWCNT composites and Ag-doped composites. (a) PFDD samples. (b) PFBPy samples, the SWCNT composite and its Ag-doped composite have a slightly smaller FWHM (0.90 vs. 1.00 eV), indicating the relatively high uniformity of the BPy conformation on the SWCNT surface. (c) The difference between PFDD and PFBPy; the red curve is the result of subtracting the PFDD curve from the PFBPy curve, and the resulting peak at 285.8 eV is attributed to the BPy unit.

6. HRTEM analysis of the Ag–PFBPy/SWCNT composite

Figure S6 displays a high-resolution conventional HRTEM image of the Ag–PFBPy/SWCNT composite sample, where a small bundle with two nanotubes and an AgNP with a diameter of ~3 nm can be seen, although particles of this size were rare in the sample. Atomic fringes (inset of Figure S6) can be seen in this particle, indicating a metallic crystal structure. This image also

shows a very thin polymer layer on the nanotube surface, even on the interface between the two nanotubes.

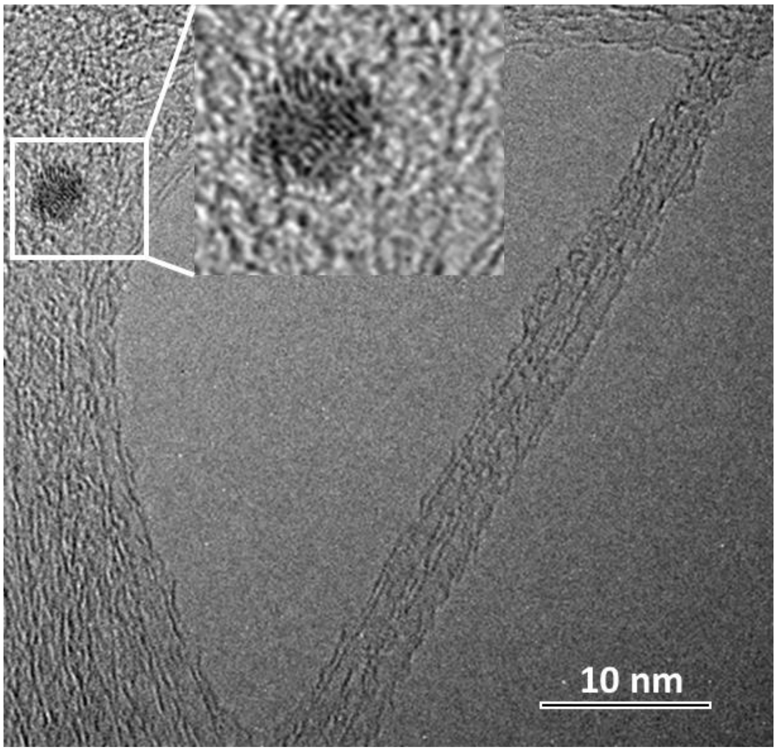

Figure S6. Conventional HRTEM image of the Ag–PFBPy/SWCNT composite, where a large AgNP of ~3 nm size and a twin SWCNT bundle were observed. A polymer layer on the nanotube can be clearly seen. The atomic fringes of the AgNP appeared clearly in the inset, indicating a crystal structure of the nanoparticles.

Figure S7 compares the ADF images of three Ag–PFBPy/SWCNT samples with the Ag^+ content increased from 0.4 to 5.0 and 50 of [Ag]/[BPy]. Figure S7a shows that whereas the average size of the AgNPs in the chain is about 0.6 nm, the size of the NPs in the other region is only approximated half of that. This is because the chain forms in a region with a high Ag^+ concentration close to the Ag–OTf droplet. However, 0.3 nm is still larger than an Ag atom, indicating that the Ag reduction preferentially occurred on the formed AgNPs to increase their size. As Ag–OTf usage

increased, particle size grew to ~0.6 nm at [Ag]/[BPy] of 5.0 (b), and further to ~0.8 at [Ag]/[BPy] of 50 (c), although with a bimodal size distribution in (c), which comprises a tiny percentage of NPs with an average size of 2.7 nm. This finding revealed that the size of the produced AgNPs in the PFBPy/SWCNT solution could be easily regulated at sub-nm level by applying a low Ag–OTf usage.

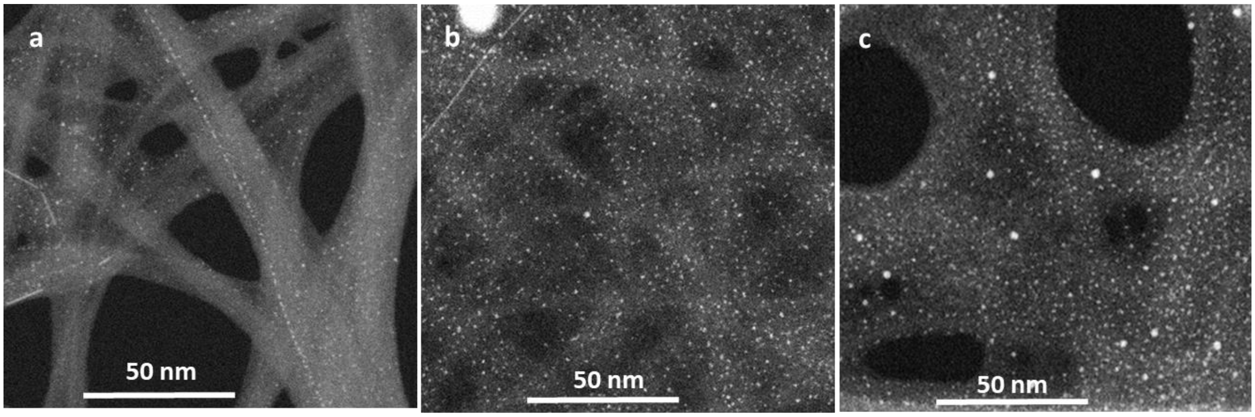

Figure S7. ADF-STEM image of the Ag–PFBPy/SWCNT composite deposited on a Lacey carbon film-coated TEM grid at [Ag]/[BPy] ratio of 0.4 (a), 5.0 (b), and 50 (c), indicating an average Ag particle size of 0.6, 1.0, and 1.3 nm. Small percentage of larger particles with size of ~2.7 nm were also observed in (c).

7. Improvement of humidity sensing capability of AgNP-doped PFBPy/SWCNT composite

Figure 8 shows that the effect of AgNP doping on the transfer curves of PFBPy/SWCNT and PFDD/SWCNT devices in N_2 is completely different. This is due to the differential in energy level between AgNP and SWCNT in these two samples. Figure S8 compares the Fermi level of AgNPs to that of SWCNTs for these two AgNP composites. In general, NPs of metal have a higher work function than polycrystalline metals.[6] The work function of AgNPs has been reported over a wide range; for example, for a particle size of ~5 nm, the reported work function values vary between 4.09 and 5.50 eV, depending on the type of ligands on the surface and the detection methods.[7-9] Therefore, in this discussion, we estimate this value based on our TFT study. As shown in Figure

8a, the Ag–PFBPy/SWCNT device in dry N_2 has a nearly identical transfer curve to the device without AgNPs, showing that there is no discernible charge transfer between the AgNPs and the SWCNTs in this device. This result indicates that the AgNPs in this device have the same Fermi level as the SWCNTs.[10] Considering that the PFBPy/SWCNT composite has nearly symmetric ambipolar n-/p-branches in its transfer curve, the intrinsic Fermi level (E_i) can be estimated as the middle value of the V1 and C1 of the SWCNTs, which are reported to be 5.10 and 4.25 eV, respectively, for the laser tube with an average diameter of 1.3 nm used in this study.[1,11,12] It results in a Fermi energy of 4,68 eV for the AgNPs. This high Fermi energy is attributed to the extremely small size of the AgNPs (~0.3 nm) and the $^{-}$OTf ligand adsorption.[7-9] This can be proven by comparing with the TFT performance of the Ag–PFDD/SWCNT device. Figure 8b demonstrates that once AgNPs were introduced into the PFDD/SWCNT composite, the material changed from ambipolar to n-doped. Figure 5 of the HRTEM data demonstrates that the AgNPs in this composite were significantly larger (1~5 nm vs. 0.3 nm). It will result in an ~0.2 eV change in the work function of the AgNPs compared to the PFBPy samples,[7] which will drop the Fermi level from 4.68 to 4.48 eV. The equilibrium of the Fermi level of AgNPs and SWCNTs in this composite led to electron flow from AgNPs to SWCNTs to n-dope the SWCNTs in this device, as indicated in Figure S8b.

Figure 8 shows that the transfer curves of both devices in ambient air are entirely distinct from those in N_2. Both materials exhibited typical p-type behavior, indicating that they are extensively p-doped. This behavior is widely observed in SWCNT-based TFT devices because the nanotubes are doped by the O_2/H_2O redox pair in the air.[1,13,14] Figure 8 demonstrated that Ag doping marginally increased the p-current in the PFBPy composite and marginally decreased the p-current in the PFDD composite. This is attributed to the combined effect of the Fermi-level alignment between AgNPs and SWCNTs and the O_2/H_2O redox doping, as illustrated in Figure S8c. It should be emphasized that although the air doping effect dominated the effect of the Fermi-level alignment in this situation due to the inexhaustible supply of air and a limited amount of charge flow from the AgNPs because of the low content of AgNPs (~4 wt%), the Fermi-level alignment nevertheless played a significant role in sensing (Figure S8c).

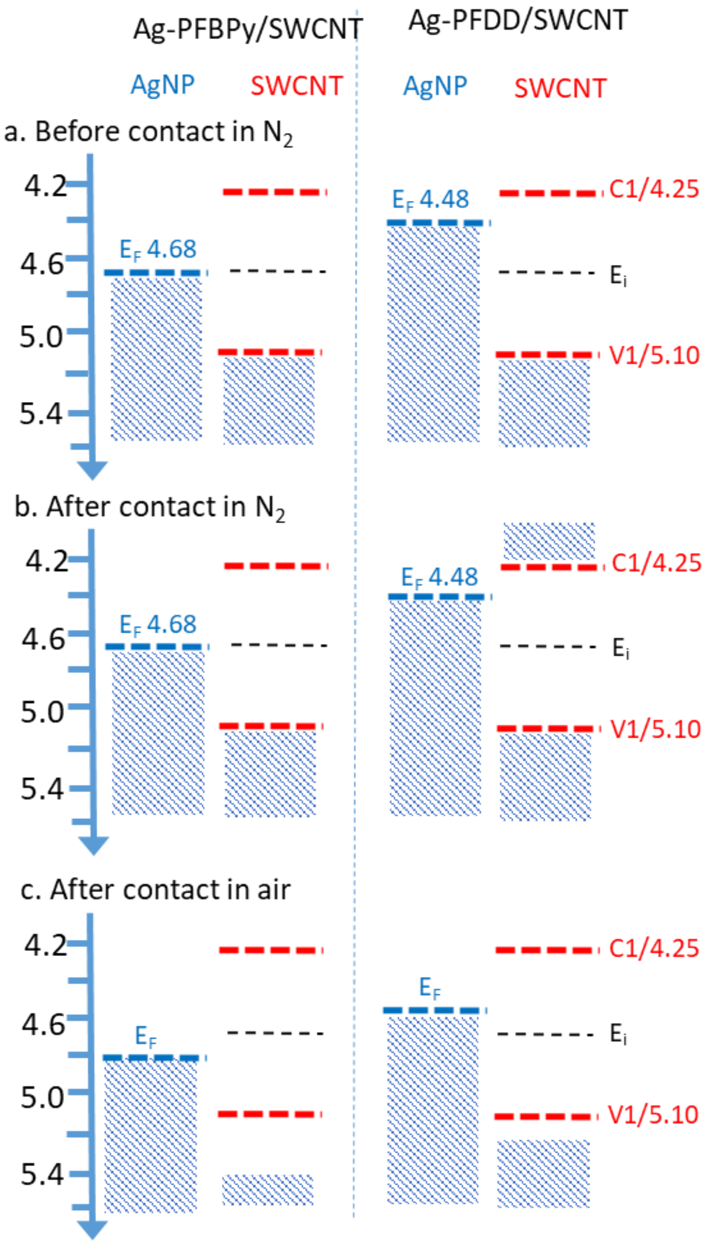

Figure S8. Energy diagram of AgNPs and SWCNTs in the Ag–PFBPy/SWCNT and Ag–PFDD/SWCNT composites before and after contact and after exposure to wet air. (E_F: Fermi energy level, E_i, intrinsic Fermi level of SWCNT). (a) The Fermi level of the AgNPs in Ag–PFBPy/SWCNT is at the same level as E_i because there is no charge flow between the AgNPs and

SWCNTs, while it is ~0.2 eV lower in the Ag–PFDD/SWCNTs due to the larger AgNP size. (b) After contact, no charge flow between the AgNPs and SWCNTs was observed in the PFBPy composite, while electron flow from the AgNPs caused the n-type behavior of the SWCNTs in the PFDD composite. (c) When exposed to wet air, SWCNT doping was dominated by the O_2/H_2O redox process, and Fermi level alignment only slightly modulated the TFT performance of the SWCNTs.

References:

1. Ding, J.; Li, Z.; Lefebvre, J.; Du X.; Malenfant, P. R. L.; Mechanistic Consideration of pH Effect on the Enrichment of Semiconducting SWCNTs by Conjugated Polymer Extraction. *J. Phys. Chem. C,* **2016**, *120*, 21946-21954.
2. Nakamoto, K.; Ultraviolet Spectra and Strucutres of 2,2’-Bipyridine and 2,2’2”-Terpyridine in Aqueous Solution. *J. Phys. Chem.,* **1960**, *64*, 1420–1425.
3. Nosek, M.; Sainio, J.; Joensuu, P. M.; 2,2’-Bipyridine-functionalized Single-walled Carbon Nanotubes: The Formation of Transition Metal Complexes and Their Charge Transfer Effects. *Carbon,* **2018**, *129,* 175-182.
4. Okpalugo, T. I. T.; Papakonstantinou, P.; Murphy, H.; McLaughlin, J.; Brown, N. M. D.; High Resolution XPS Characterization of Chemical Functionalised MWCNTs and SWCNTs. *Carbon,* **2005**, *43*, 153–161.
5. Alemán, B.; Vila M.; Vilatela, J. J. Advanced surface chemistry analysis of carbon nanotube fibers by X-ray photoelectron spectroscopy. *Phys. Status Solidi A*, **2018**, *215*, 1800187.
6. Plieth, W. J. The work function of small metal particles and its relation to electrochemical properties. *Surf. Sci.,* **1985**, *156*, 530–535.
7. Schnippering, M.; Carrara, M.; Foelske, A.; Kotz, R.; Fermın, D. J. Electronic properties of Ag nanoparticle arrays. A Kelvin probe and high resolution XPS study. *Phys. Chem. Chem. Phys.*, **2007**, *9*, 725–730.
8. Seong, M.; Kim, H,; Lee, S.; Kim D.; Oh, S. J. Engineering the work function of solution processed electrodes of silver nanocrystal thin film through surface chemistry modification. *APL Mater.*, **2018**, *6*, 121105.

9. Wang, P.; Tanaka, D.; Ryuzaki, S.; Araki, S.; Okamoto, K.; Tamada, K. Silver nanoparticles with tunable work functions. *Appl. Phys. Lett.*, **2015**, *107*, 151601.
10. Kao, K. C. Charge Carrier Injection from Electrical Contacts, in “Dielectric Phenomena in Solids. *Mater. Sci.*, **2004**, 327-380.
11. Chakrapani, V.; Angus, J. C.; Anderson, A. B.; Wolter, S. D.; Stoner, B. R.;Sumanasekera, G. U. Charge Transfer Equilibria Between Diamond and an Aqueous Oxygen Electrochemical Redox Couple. *Science*, **2007**, *318*, 1424−1430.
12. Kim, K. K.; Yoon, S.-M.; Park, H. K.; Shin, H.-J.; Kim, S. M. Bae, J. J.; Cui, Y.; Kim, J. M.; Choi, J.-Y.; Lee, Y. H. Doping Strategy of Carbon Nanotubes with Redox Chemistry. *New J. Chem.*, **2010**, *34*, 2183−2188.
13. Aguirre, C. M.; Levesque, P. L.; Paillet, M.; Lapointe, F.; St-Antoine, B. C.; Desjardins, P.; Martel, R. The Role of the Oxygen/Water Redox Couple in Suppressing Electron Conduction in Field-Effect Transistors. *Adv. Mater*. **2009**, *21*, 3087−3091.
14. Chakrapani, V.; Angus, J. C.; Anderson, A. B.; Wolter, S. D.; Stoner, B. R.; Sumanasekera, G. U. Charge transfer equilibria between diamond and an aqueous oxygen electrochemical redox couple. *Science,* **2007**, *318*, 1424−1430.
